# Endoplasmic Reticulum Geometry Dictates Neuronal Bursting via Calcium Store Refill Rates and Exposes Selective Neuronal Vulnerability

**DOI:** 10.1002/advs.202521101

**Published:** 2026-03-31

**Authors:** Valentina Davi, Pierre Parutto, Yuyi Zhang, Tasuku Konno, Cecile Crapart, Raquel Pereira, John P. Franklin, Mosab Ali Awadelkareem, Daniel C Maddison, Michael J. Devine, Edgar R. Gomes, Joseph Chambers, Elena Koslover, Edward Avezov

**Affiliations:** ^1^ Department of Clinical Neurosciences UK Dementia Research Institute at University of Cambridge Cambridge UK; ^2^ Department of Physics University of California San Diego, La Jolla California USA; ^3^ GIMM – Gulbenkian Institute for Molecular Medicine Avenida Prof. Egas Moniz Lisboa Portugal; ^4^ Faculdade de Medicina Universidade De Lisboa Av. Prof. Egas Moniz Lisboa Portugal; ^5^ Mitochondrial Neurobiology Laboratory The Francis Crick Institute London UK; ^6^ Department of Basic and Clinical Neuroscience Institute of Psychiatry Psychology and Neuroscience King's College London London UK; ^7^ Department of Clinical and Movement Neurosciences UCL Queen Square Institute of Neurology University College London London UK; ^8^ Cambridge Institute for Medical Research (CIMR) Department of Medicine University of Cambridge Cambridge UK; ^9^ Nuffield Department of Clinical Neurosciences University of Oxford Oxford UK

**Keywords:** calcium oscillations, endoplasmic reticulum Ca^2+^ refill, endoplasmic reticulum morphology, human iPSC‐derived neurons, modelling, neurodegenerative diseases, neuronal firing

## Abstract

The endoplasmic reticulum (ER)’s continuous morphology is tightly controlled by ER‐shaping proteins, whose genetic or expression defects drive a spectrum of neurodegenerative disorders from Hereditary Spastic Paraplegia to Alzheimer's disease. Why perturbations in ER morphology manifest specifically in neurons remains unknown. Here, by coupling visualisation of global sub‐Hz firing bursts to ER ultrastructural manipulations in human inducible Pluripotent Stem Cells (hiPSC)‐derived cortical neurons, alongside physical simulations, we establish a key ER structure‐function principle: neuronal ER architecture dictates Ca^2+^ replenishment speed. Altering ER structure hinders network ER luminal connectivity and Ca^2+^ propagation from refill points at plasma membrane contact sites, impairing the ER's capability to supply repetitive Ca^2+^ bursts. The ER morpho‐regulatory control of Ca^2+^ refill speed thus constitutes a switch on neuronal activity. Further, perturbed ER shape also abolishes Ca^2+^ firing and contraction in primary skeletal muscle cells. These results expose the selective vulnerability of Ca^2+^‐firing cells to ER structural disruptions, rationalizing ER dysfunction in neurodegeneration and unveiling a new role for the continuous ER morphology that could apply universally to Ca^2+^‐firing cells.

## Graphical abstract

The ER's continuous tubular network is maintained by ER‐shaping proteins whose mutation or dysregulation contributes to neurodegenerative diseases. Here, we show that ER morphology sets the speed of Ca^2^^+^ store replenishment between firing events. Disrupting ER continuity slows intra‐ER Ca^2^^+^ redistribution from extracellular refill (SOCE) sites, driving burst rundown and functional failure—offering a mechanism for selective neuronal vulnerability to ER defects.

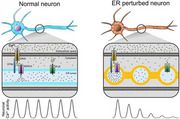

## 1 Introduction

The endoplasmic reticulum is the only organelle that spans an entire neuron—threading soma, dendrites and axons as one contiguous network of 30–80 nm‐wide tubules [1, 2, 3]. The membrane‐curvature factors that sculpt this architecture — reticulons, REEPs, atlastins, and spastin [4, 5, 6] — carry pathogenic mutations that cause Hereditary Spastic Paraplegia (HSP) [7, 8, 9] and recur as de‐novo variants or mis‐expressed genes in other neurodegenerative pathologies including Amyotrophic Lateral Sclerosis (ALS) [10, 11, 12] and Alzheimer's disease (AD) [12, 13, 14]. Although these ER‐morphogens are expressed across tissues, manifestation of their disruption concentrates in neurons. This raises the question of what unique functional feature of ER architecture makes neurons so vulnerable to its perturbation.

The ER's continuous lumen allows soluble cargos — ions, metabolites, chaperones — to move rapidly between distant cellular regions [15, 16]. Among those cargos, Ca^2^^+^ is extraordinary: the ER maintains Ca^2^^+^ concentrations four orders of magnitude higher than the bordering cytoplasm — in the form of sub‐millimolar free Ca^2^^+^, and about twentyfold equivalent bound to the protein Ca^2+^ capacitors, buffering its concentration. These endow the ER with both the depth and mobility of stores needed to operate as the cell's dominant internal Ca^2+^ handling system [17]. Ca^2^^+^ is a universal second messenger, and neurons exploit it to control electrophysiological signalling [18]. We therefore postulate that the ER's continuous tubular lattice is optimised for rapid mobility of luminal solute, and that disrupting this architecture would cripple Ca^2^^+^ mobilisation, disproportionately affecting cells with expansive geometry and relying on frequent Ca^2+^ transients. Thus, preserving tubular continuity would be critical for neurons because it enables swift, long‐range redistribution of luminal Ca^2^^+^ (and other contents) to sites of demand.

Investigating the plausibility of this idea first requires determining whether and how neurons deploy ER Ca^2^^+^ during neurophysiological activities. Neuronal Ca^2^^+^ signalling spans two extremes: millisecond nanodomains beneath pre‐ and post‐synaptic zones arise from Ca^2+^ influx via the plasma membrane [19]. ER participation in these fast, low‐amplitude events is partial: store depletion can leave short‐term synaptic plasticity and action potential‐induced Ca^2+^ transients intact [20, 21], whereas other work reports that ER presence correlates with boosts of spine transients and synaptic strengths [22, 23, 24, 25, 26]. At the opposite end, seconds‐long, high‐amplitude Ca^2+^ waves that accompany electrophysiological bursts recur at sub‐hertz frequencies during cortical development [27, 28] and in coordinated regional activities in adult brains [29, 30, 31, 32]. In theory, a sustained influx of Ca^2+^ down the gradient across the plasma membrane alone could fuel such global events and whether the ER contributes to them is unknown. Other excitable tissues with comparable time scale and magnitude firing involve the ER: myocytes’ ER (sarcoplasmic reticulum) is indispensable for excitation–contraction in cardiac and skeletal muscle [33, 34, 35], and the ER also supports astrocytes’ Ca^2^^+^ waves [36].

Here, we combine orthogonal perturbations of ER morphology with Ca^2^^+^ imaging in human cortical neurons, supported by an in‐silico model of ER architecture and its contribution to neurophysiology. We find that the ER critically fuels sub‐hertz neuronal network bursts with a supply of Ca^2+^. Slowing intra‐ER Ca^2^^+^ carrier‐protein mobility via orthogonal manipulations to tubular continuity disrupts the normal firing regime. Studying the refill kinetics dependence on ER structural integrity revealed that interrupting the tubular continuity doubles the half‐time of Ca^2^^+^ store refill from plasma membrane (PM)‐ER contact sites, which are necessary for its replenishment after a firing event. This leaves the lumen under‐supplied and unable to keep up with Ca^2+^ demand during repetitive activity, thus abolishing the sub‐Hz, high‐amplitude network bursts. The bursts’ perturbation thus occurs even though fast local Ca^2+^ transients of synaptic activity, fuelled by the extracellular pool, can function normally, and the neurons are still excitable. Perturbing ER shape also suppressed Ca^2+^ transients and contractions in primary skeletal muscle cells. Therefore, the principle of the ER architecture's importance in sustaining timely Ca^2+^ resupply during firing could be applicable to any other tissue using global firing for function, including astroglia (known for similar scale ER Ca^2+^ firing [33, 36]).

## 2 Results

### 2.1 ER Ca^2^^+^ Release via IP_3_ Receptors Fuels Synchronous Network Bursts in Human iPSC‐Derived Cortical Neurons

**FIGURE 1:**
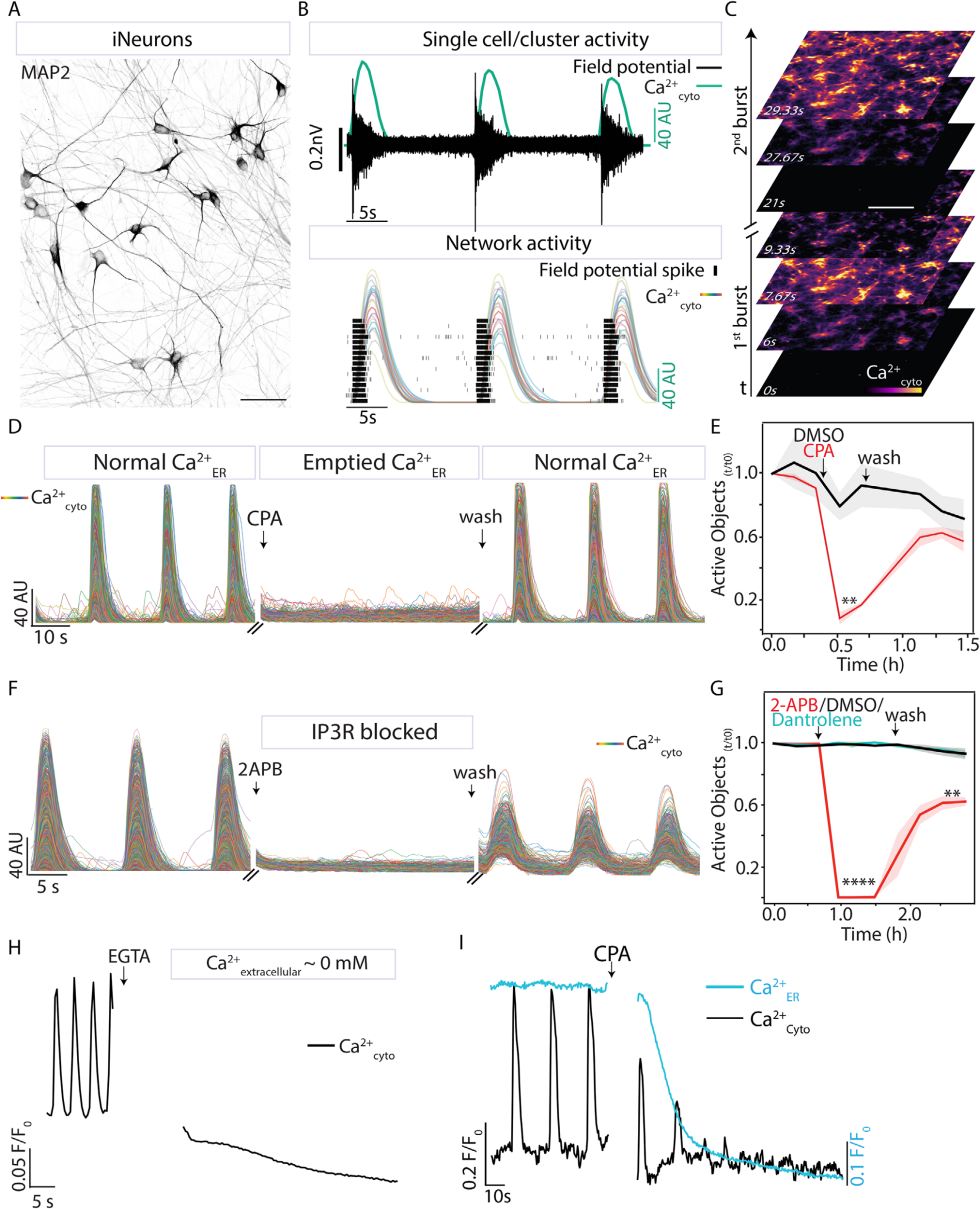
iNeurons’ network Activity Is Fuelled by ER Ca^2+^ in an IP3R‐dependent Manner. (A) Micrograph of iNeurons after 21 days of differentiation, stained with microtubule‐associated protein 2 (MAP2, neuronal marker). Scale bar: 50 µm. (B) Field potential detected from a single electrode of a Multi‐Electrodes Array (MEA) over time in iNeurons (top) and temporal raster plot of detected spikes from multiple electrodes (bottom), both overlayed with single iNeurons’ cytosolic Ca^2+^ traces (detected as in C). (C) Time series of cytosolic Ca^2+^ detected by an mRuby‐based Ca^2+^ sensor (Neuroburst) in Day 30 iNeurons differentiated as in (A). Scale bar: 200 µm. (D) Single‐cell cytosolic Ca^2+^ traces detected as in (C) from a representative culture before, 1 min after CPA treatment (SERCA pump blocker, 25 µm), and 5 min after wash‐out. (E) Quantification of firing cells (Active Objects) normalized to T_0_ over time in CPA (red) and DMSO (black) treated cultures (solid line: mean, shade: STD, *n* = 3 wells). (F) Single‐cell cytosolic Ca^2+^ traces detected as in (C) treated with 2‐APB (IP3R blocker, 10 µm) as in (D). (G) Quantification of firing cells as in (E) in 2‐APB (red), Dantrolene (cyan, 10 µm) and DMSO (black)‐treated cultures (solid line: mean, shade: STD, *n* = 3 wells). (H) Cytosolic Ca^2+^ trace detected as in (C) during treatment with EGTA (3 mm). (I) Cytosolic Ca^2+^ (black) and ER Ca^2+^ (cyan) traces simultaneously detected through Neuroburst and ER‐GCaMP6_150_ respectively during CPA (25 µm) treatment. ^**^ *p* < 0.01, ^****^ *p* <0.0001 from Student's *t*‐test.

Synchronous network bursts at sub‐Hz scale appear initially in developing cortical circuits [27, 28] and locally in adults (e.g. in visual cortex and hippocampus’ dentate gyrus) [29, 30, 31, 32]. To establish a quantifiable read‐out of this activity, we differentiated NGN2‐induced human cortical neurons [37] (*iNeurons*, Figure 1A) to a point when the cultures formed synaptically connected networks that generated culture‐wide high‐amplitude slow Ca^2^^+^ transients (mean duration (full width at half maximum)   =  2.5 ± 0.5 s, inter‐burst interval  =  15.8 ±  3.8 s, Figure 1B,C; Video S1). Each optical Ca^2+^ burst mirrored action‐potential trains detectable by multielectrode arrays (Figure 1B). The addition of a voltage‐gated sodium channel blocker, Tetrodotoxin (TTX) or synaptic inhibitors — the AMPA/NMDA blockers CNQX + MK‐801 — abolished the Ca^2+^ bursts (Figure S1A–D), confirming that the readout reflects canonical, action‐potential‐driven network activity. Its measurable parameters — synchrony, amplitude, and frequency — enable determining the impact of specific and manipulatable organelle biology aspects on neuronal activity.

Using this system, we first asked whether intracellular Ca^2^^+^ stores support these high‐amplitude, slow (compared to the millisecond‐scale local synaptic transients) and coordinated bursts. The millimolar extracellular Ca^2^^+^ concentration is sufficient in principle to explain the cytosolic rise. However, ER Ca^2+^ contributes to global waves in muscle and glia cells [34, 35, 36, 38]. Therefore, it stands to reason that the system controlling neuronal firings on a similar space‐time scale is also designed to draw on ER‐supplied Ca^2+^. Indeed, depleting the ER Ca^2+^ by irreversible inhibition of SERCA – the pump responsible for ER Ca^2+^ re‐uptake from the cytosol – through Thapsigargin (TG) stopped Ca^2+^ firing (Figure S1E,F). A reversible SERCA inhibitor, cyclopiazonic acid (CPA) caused an identical quenching of burst activity paralleled by a reduced ER Ca^2+^ concentration, but this time the bursts recovered immediately after the drug wash‐out and ER Ca^2+^ replenishment (Figure 1D,E,I; Video S2). A Ca^2^^+^‐loaded ER is therefore indispensable for these global network events. This conclusion is strengthened by the sensitivity of the neurons’ firing ability to blocking one of the ER Ca^2+^ release channels – IP_3_‐receptor – with its antagonist 2‐APB. The drug eliminated Ca^2+^ firing with the same speed and completeness as SERCA inhibition (Figure 1F,G), whereas the ryanodine‐receptor (RyR) blocker Dantrolene had no effect (Figure 1G; Figure S1G). This result is consistent with IP3‐dependency of Ca^2+^ waves in other neuronal subtypes, and the absence of reports of RyRs‐mediated Ca^2+^ waves in neurons [39].

Further, Ca^2+^ bursts were also abolished by upstream blockage of the IP3 pathway by pharmacological inhibition of either Phospholipase C (PLC) or group 1 metabotropic glutamate receptors (mGluRs, Figure S1H–K). Therefore, as ER Ca^2+^ release (triggered by mGluRs‐IP3 cascade), is a result of glutamatergic synaptic transmission, the burst abolishment after CPA is caused by emptying of the ER stores while firing under replenishment blockage. Notably, chelating extracellular Ca^2^^+^ with 3 mm EGTA instantaneously terminated bursts (Figure 1H), contrasting with a gradual run‐down of burst amplitude caused by blocking the ER‐specific uptake via SERCA inhibition (Figure 1I). This is consistent with a two‐step causal chain — extracellular Ca^2^^+^ influx triggering synaptic vesicles release, leading to activation of mGluRs and IP_3_R‐gated ER Ca^2+^ release to sustain high‐amplitude, durable bursts.

Depletion of ER Ca^2^^+^ has been reported to be inert for or even increase vesicle release [40]. Thus, we presume that the ER Ca^2+^ is not a prerequisite for the fast local Ca^2+^ transients of synaptic releases. These may solely rely on the flow of the ion across plasma membrane from the high‐Ca^2+^ extracellular milieu. On the other hand, the ER is indispensable for sustaining the global seconds‐long Ca^2^^+^ bursts. The dependence of the neuronal network activity on ER Ca^2+^ release establishes it as the quantitative baseline for testing if and how ER geometry influences neuronal behavior.

### 2.2 ER Fragmentation Abolishes Synchronous Network Firing

**FIGURE 2:**
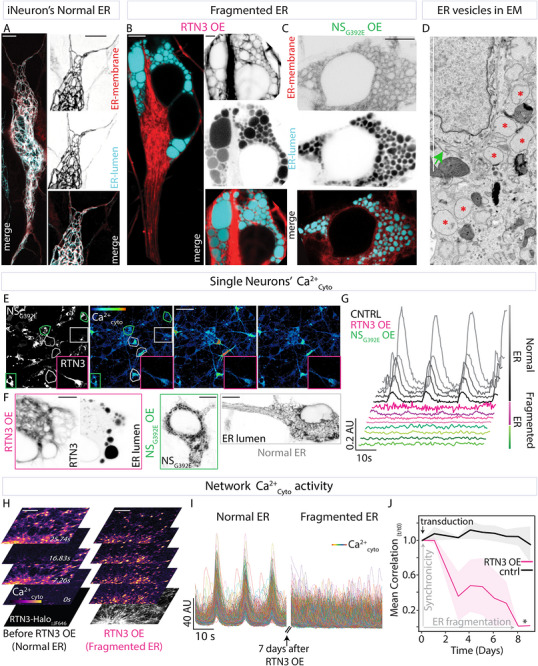
ER With Perturbed Morphology Fails to Support iNeurons’ cytosolic Ca^2+^ Bursts (A–C). Micrographs of the ER in iNeurons with exogenously expressed: (A) ER membrane protein Halo‐Sec61β labelled with JF646 (red) and ER luminal marker mEmerald‐KDEL (cyan), (B) ER membrane protein RTN3a‐Halo labeled with JF646 (red) and ER luminal marker D4ER (cyan), (C) Halo‐NS_G392E_ labeled with JF646 (cyan) and ER membrane marker ER‐targeted GCaMP3 (red). Note the accumulation of ER luminal content in vesicles in (B) and (C). (D) Scanning electron micrograph of iNeurons with deformed ER as in (C) (Red stars: ER vesicles, green arrow: normal ER). Scale bars (A–D): 5 µm. (E) Micrographs of iNeurons with sparse exogenous expression of Halo‐NS_G392E_ or RTN3a‐Halo (inset), both labelled with JF650 (left) and time series of the Ca^2+^_cyto_ detected through Neuroburst in the same field of view (right). Magenta Region of Interest (ROI): RTN3OE cell, Green ROIs: NS_G392E_OE cells, Gray ROIs: controls (not infected). Scale bar: 100 µm. (F) Magnifications of rectangular ROIs in (E). RTN3 and NS_G392E_ are labelled as in (E). ER lumen is visualised by exogenous expression of ER‐mEmerald. Scale bars: 5 µm. (G) Single‐cell cytosolic Ca^2+^ traces of CNTRL (black), RTN3aOE (magenta) and NS_G392E_OE (green) iNeurons from (E). (H) Time series of Ca^2+^_cyto_ detected through Neuroburst in iNeurons before (left) and after 7 days of exogenous expression of RTN3a (right). Note the loss of synchronous activity. Scale bars: 50 µm. (I) Single‐cell cytosolic Ca^2+^ traces from (G). (J) Mean time correlation between traces as in (I) over time (magenta: RTN3a OE, black: control, not transduced). Solid line: mean, shade: STD, *n* = 3 wells, ^*^ *p* <0.05 from Student's *t*‐test.

**FIGURE 3:**
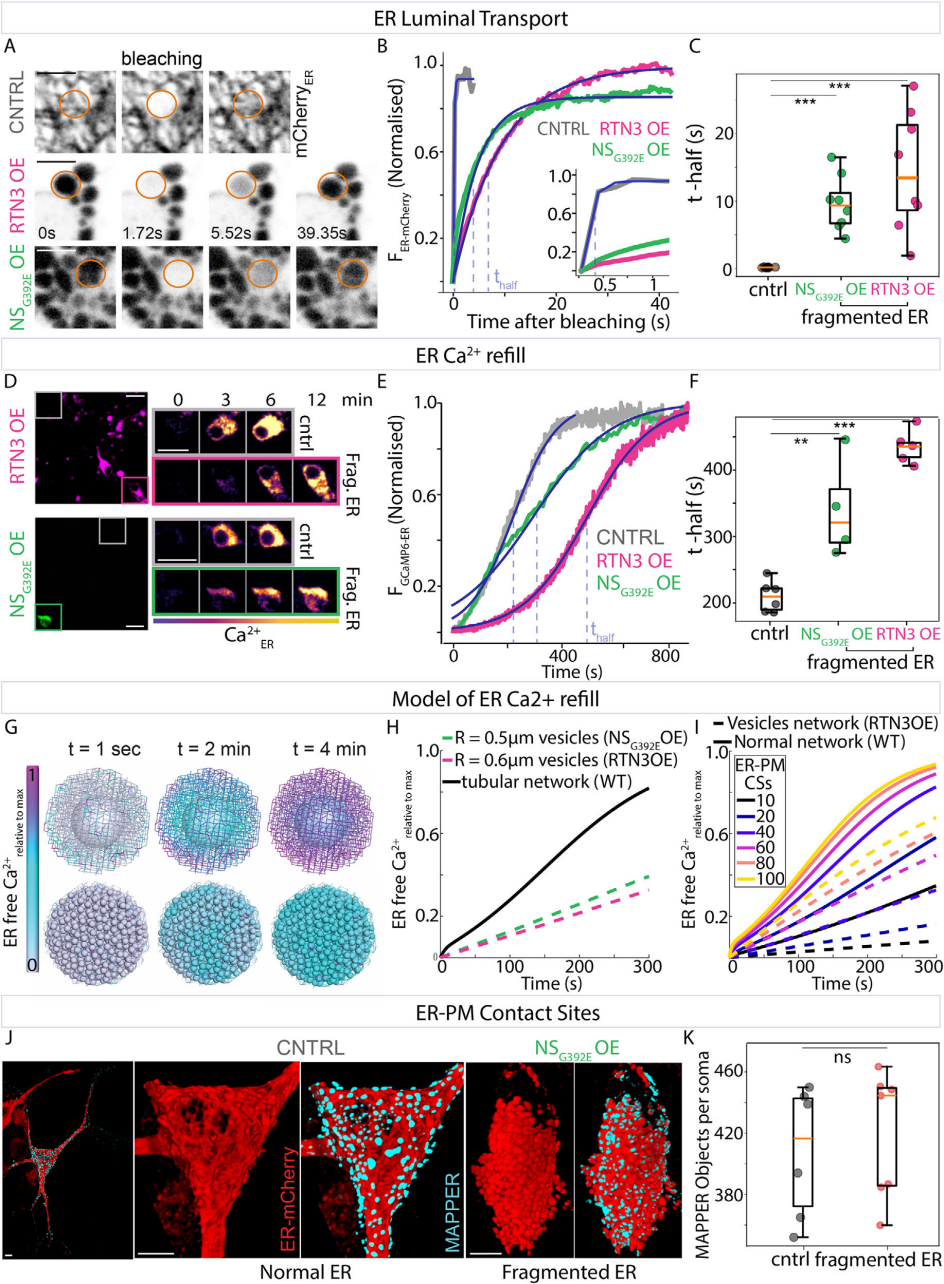
ER Structure Defines ER Ca^2+^ Refill. (A) Fluorescence Recovery After Photobleaching (FRAP) time series of the ER luminal protein mCherry‐KDEL in normal (top) and fragmented ER (RTN3aOE middle, NS_G392E_OE bottom) in iNeurons. The orange circles represent the bleached area. Scale bars: 2 µm. (B) mCherry‐KDEL intensity traces after photobleaching as in (A) (black: control (not infected), magenta: RTN3aOE, green: NS_G392E_OE). Blue curve: exponential fit, light blue dashed line: half recovery time (t‐half). Inset: blow‐up of the same plot. (C) Half recovery time (t‐half) values from exponential fits as in (B) from WT (*n* = 8 cells), RTN3 OE (*n* = 8 cells, ^***^ *p* = 4.43 × 10^−4^), and NS_G392E_OE (*n* = 8 cells, ^***^ *p* = 1.16 × 10^−5^) cells. Each dot represents the t‐half from a single cell. (D) Micrographs of iNeurons with sparse exogenous expression of RTN3a‐Halo (top, magenta) and Halo‐NS_G392E_ (bottom, green), both labelled with JF646 (left) and time series of the ER Ca^2+^ sensor GCaMP6_150_ in the same field of view (right) after washout of BTP2 (ORAI1 blocker, 10 µm, 20 min incubation). Magenta ROI: RTN3aOE cell, Green ROI: NS_G392E_OE cell, Gray ROIs: controls (not infected). Scale bars: 20 µm. (E) Single‐cell ER Ca^2+^ trace detected and treated as in (D) of control (black), RTN3aOE (magenta) and NS_G392E_OE (green) iNeurons. Blue curve: sigmoid fit, light blue dashed line: time to half‐recovery (t‐half). (F) T‐half values from sigmoid fits as in (E) of control (*n* = 6 cells), RTN3aOE (*n* = 5 cells, ^***^ *p* = 1.48 × 10^−7^) and NS_G392E_OE (*n* = 4 cells, ^**^ *p* = 5.49 × 10^−3^) iNeurons. Each dot represents the t‐half from a single cell. (G) Simulation snapshots of Ca^2+^ refill from ER‐PM contact sites. Color corresponds to free luminal Ca^2+^ levels, at increasing times after refill is initiated. Top: tubular network, bottom: partially fragmented network of spherical vesicles of 0.6 µm radius connected by narrow tubes. (H) Average free luminal Ca^2+^ throughout the network, in the spatial transport model, plotted against simulation time. Black solid line: tubular network. Dashed lines: networks of spherical vesicles with radius 0.6 µm (pink) and 0.5 µm (green), representing RTN3aOE and NS_G392E_OE ER structures, respectively. (I) Average luminal free Ca^2+^ plotted over time for simulations with the tubular network (solid lines) and spherical vesicle network (dashed lines). Colors correspond to different number of ER‐PM contact sites with fixed unbound Ca^2+^ concentration. (J) 3D reconstructions from confocal z‐stacks of mCherry‐KDEL (red) and MAPPER (cyan) in WT (left) and NS_G392E_OE (right) iNeurons. Scale bars: 5 µm. (K) MAPPER clusters per cell detected as in (J) in iNeurons with normal (*n* = 6) and fragmented ER (NS_G392E_OE and RTN3aOE, *n* = 6 cells, ^ns^ *p* = 0.9). Each dot represents MAPPER clusters from a single cell. Statistical significance was determined by Student's *t*‐test.

Having documented that ER Ca^2+^ fuels global firing, we next examined whether the continuous tubular architecture of the organelle is required for this neuronal activity. We perturbed ER morphology in two orthogonal ways: first, we overexpressed the short isoform of the ER membrane protein Reticulon 3 (RTN3a), which leads to ER vesiculation while avoiding any indirect effect from its functional cytosolic tail, which acts as an ER‐phagy adaptor [41] (Figure 2B). Second, we attained a similar structural ER perturbation by orthogonal means, this time provoking it from within the ER lumen. We overexpressed the dementia‐linked mutant Neuroserpin (NS_G392E_, cause of Familial encephalopathy with neuroserpin inclusion bodies—FENIB) [42, 43], to induce ER vesiculation via protein accumulation in the lumen (Figure 2C) – an ER distortion similar to that caused by its analogous serpin, α1‐antitrypsin [44]. We used these manipulations to convert the normal reticular network (Figure 2A) into vesicular, scantily interconnected ER‐clusters — as visualised by live‐cell fluorescence (Figure 2B,C) and scanning electron microscopy (SEM) of iNeurons (Figure 2D; for assessment of luminal connectivity between clusters, see Figure 3A–C). Expression of these constructs was induced in cells with fully formed neurites (after Day 14), to avoid any potential effect of ER misshaping on neuronal development. The neurites appeared preserved in cells with high expression of the ER‐perturbing agents (Figure S2A,D). These manipulations left viability apparently unchanged, at least within the timeframe of subsequent experiments (Figure S2A,B).

Further, ER structural disturbance by RTN3 did not cause ER stress: levels of messenger RNA of *CHOP* and percentage of spliced *XBP1* (reflecting respective activation of the PERK and IRE1 arms of the Unfolded Protein Response, UPR [45]) were unchanged in the morphogen overexpressing neurons. NS_G392E_ also did not triggerXBP1 marker but caused a mild increase in CHOP (30% the effect size of pharmacological stress characteristically induced by Thapsigargin, Figure S2E–G). Given that one of the manipulators is UPR‐inert and the other partially stressing but both share the morphogenic effect, the phenotypes they may induce could be attributable to ER membrane misshaping, independent of ER stress.

Strikingly, within the synchronously firing networks, iNeurons with ER fragmentation induced by either misshaping factor were not active (Figure 2E–G; Videos S3 and S4). In contrast, neighbouring cells that did not express the transgenes in the same field retained normal Ca^2+^ firing (Figure 2E–G). At the culture level, the mean correlation coefficient—a proxy for network synchrony—declined steadily after RTN3a overexpression in the majority of cells (Figure 2H–J; Video S5). Since the same burst‐cancelling effect was attainable through two independent manipulations, similar only in their capacity to perturb ER morphology, it is reasonable to rule out the possibility that their other functions are responsible for this effect. Thus, we conclude that the loss of ER continuity prevents single‐neuron Ca^2+^ firing and abolishes synchronous network firing.

### 2.3 ER Fragmentation Hinders Intra‐ER Ca^2^^+^ Transport, Slowing Store Refill

Next, we investigated the mechanism underlying the absence of spontaneous cytosolic bursts in iNeurons with altered ER morphology. ER free and total Ca^2+^ remain within the normal range upon ER‐fragmentation in iNeurons, as previously observed in COS‐7 cells [15] (free ER Ca^2+^ concentration in absolute terms was measured by calibrated fluorescence life time imaging, FLIM of D4ER‐Tq probe [15] (Figure S3A–C), while total was assessed by measuring the Tg‐releasable pool (Figure S3D,E)). Further, the amplitude and kinetics of IP_3_‐evoked Ca^2^^+^ transients in ER‐perturbed neurons were indistinguishable from wild‐type (Figure S3F,G). Therefore, the absence of bursts in iNeurons with altered ER morphology should not be due to defects in the ER's ability to store or release Ca^2+^.

This leaves the kinetic consideration of Ca^2+^ delivery to ER stores as the prime parameter suspected to be dependent on ER structural integrity. To support repeated releases, the ER Ca^2+^ pool replenishment should proceed at a speed matching demand. The ER cannot be replenished solely by SERCA‐mediated re‐uptake of Ca^2+^ back from the cytoplasm, as the ion is also rapidly cleared to the cell exterior by plasma membrane pumps. This is evident in classical experiments showing fast decay of cytoplasmic Ca^2+^ upon SERCA inhibition (e.g [46, 47, 48].). The critical contribution of the extracellular Ca^2+^ for store refill is further evident from the inability of ER to replenish post‐release when Ca^2+^ in the extracellular medium is chelated [49] (Figure S4).

Thus, we postulate that the ER refill rate depends on Ca^2+^ propagation from its intake points at ER‐PM contact sites — hubs of store‐operated Ca^2+^ entry (SOCE) from the extracellular space — to the bulk of the ER network volume. Since Ca^2+^ propagation inside the ER is partly dictated by the mobility of its high‐capacity carriers [15], we sought to assess whether the extent of reduction in protein mobility upon ER fragmentation could explain the retarded ER refill. Fluorescence Recovery After Photobleaching (FRAP) measurements showed a ∼75‐fold reduction in luminal protein mobility (Figure 3A–C, S5A,B), on par with luminal transport reduction observed in ER‐perturbed COS‐7 cells [15]. Crucially, the slower protein mobility correlated with a reduction in ER‐refill rate, as visualised by the speed of Ca^2+^ recovery after washout of ORAI1 inhibitor (BTP‐2) — which causes ER Ca^2+^ depletion by blocking of ER‐PM refill points. The wild‐type neurons’ ER Ca^2^^+^ refill rate of t_half_ = 229.5 ± 34.6 s was slowed to 434.5 ± 21.6 s by RTN3a overexpression and to 341.1 ± 66.5 s by NS_G392E_ (Figure 3D–F; Video S6). Milder perturbation of ER structure (a partial reduction in 3‐way junctions) elicited by exogenous expression of Atlastin 1 dominant negative mutant (ATL1 K80A^50^, Figure S6A) caused a modest slowdown of luminal transport (∼1.6 fold increase in FRAP t_half_ compared to ∼60 and ∼90‐fold in NS_G392E_ and RTN3, respectively (Figure S6B,C)). This effect was insufficient to cause a detectable change in refill speed (Figure S6D,E).

To correlate the slowdown in Ca^2+^ refill kinetics with perturbed ER morphology, we established an *in‐silico* physical model representing transport of Ca^2+^ through the ER network. As illustrated in Figure 3G, the wildtype ER was represented as a lattice‐like network of tubules, connected to the extracellular environment at PM contact sites and to a perinuclear reservoir. The fragmented ER structures resulting from RTN3 and NS_G392E_ overexpression were represented as a network of larger and smaller spherical vesicles, respectively, connected to nearest neighbours by narrow short tubes. The vesicles serve as traps for diffusive particles, which must find narrow tubule entrances to escape. Notably, estimates of diffusive transport imply that the effective rate of spread through the network of spheres is expected to be significantly reduced compared to a tubular network (see Modelling in Methods for details), and that larger vesicles should yield slower transport, supporting our experimental findings (Figure 3A–C).

We employed numerical simulations to explicitly compute refill rates for these distinct network structures, assuming a fixed free Ca^2+^ concentration at PM contact sites and an initially empty ER lumen. The tubular structure was found to fill substantially faster than the partially fragmented structures, with larger vesicles showing slower refill (Figure 3G,H). A larger size for the cell soma amplifies the effect of transport, leading to more extreme differences between the tubular and vesicular network (Figure S7).

These refill rates are limited by a combination of factors, including bulk transport through the 3D network (substantially slower in the vesicular structures) and the number of contacts with the plasma membrane (Figure 3I). To rule out the possibility that the impaired refill in neurons with fragmented ER was caused by disruption of ER‐PM contacts, we examined their density (visualised with MAPPER [51]) and found no change in neurons with perturbed ER (Figure 3J,K). However, since MAPPER is an exogenously expressed probe, inertness of which is presumed but may not be stringent, its potential to promote or stabilise ER‐plasma membrane contact sites cannot be completely excluded. Therefore, as an orthogonal verification of contact sites’ existence in cells with structurally manipulated ER, we examined scanning electron micrographs of neurons with vesiculated ER, in which we found that contacts were present and comparable in size to those formed in cells with unperturbed ER (Figure S8A,B). This is consistent with a previous report of contact sites persisting despite vesiculation of the ER structure [52]. Despite the similar structure and density of ER‐PM junctions, the contacts formed by fragmented ER might be functionally impaired, i.e. SOCE at ER‐PM contacts might be perturbed. To address this, we examined the functionality of SOCE at ER‐PM contact sites by canonical SOCE assessment [53]: in the absence of extracellular Ca^2+^, Thapsigargin is added to induce ER Ca^2+^ depletion. Then, Ca^2+^ is introduced to the extracellular environment, resulting in SOCE and thus Ca^2+^ entry to the cytosol. As ER uptake of this Ca^2+^ is inhibited by Thapsigargin, the resulting cytosolic Ca^2+^ spike serves as a proxy for SOCE activity. The efficiency of SOCE in neurons with fragmented ER was indistinguishable from untreated cells (Figure S8C–E). Notably, physical modelling predicted a greater sensitivity of overall refill to transport hindrance than to reduction in SOCE efficiency (Figure S9). These results indicate that the refill defect should therefore lie in Ca^2^^+^ transport within the fragmented ER rather than in SOCE engagement at the periphery.

Together, the *in‐silico* model and the experimental measurements of ER transport and refill show that a continuous ER network is required to move Ca^2^^+^ rapidly from peripheral entry points to the bulk reservoir. Thus, ER shape dictates its store refill rate.

### 2.4 Slow ER Refill Hinders Ability to Generate Persistent Bursts

**FIGURE 4:**
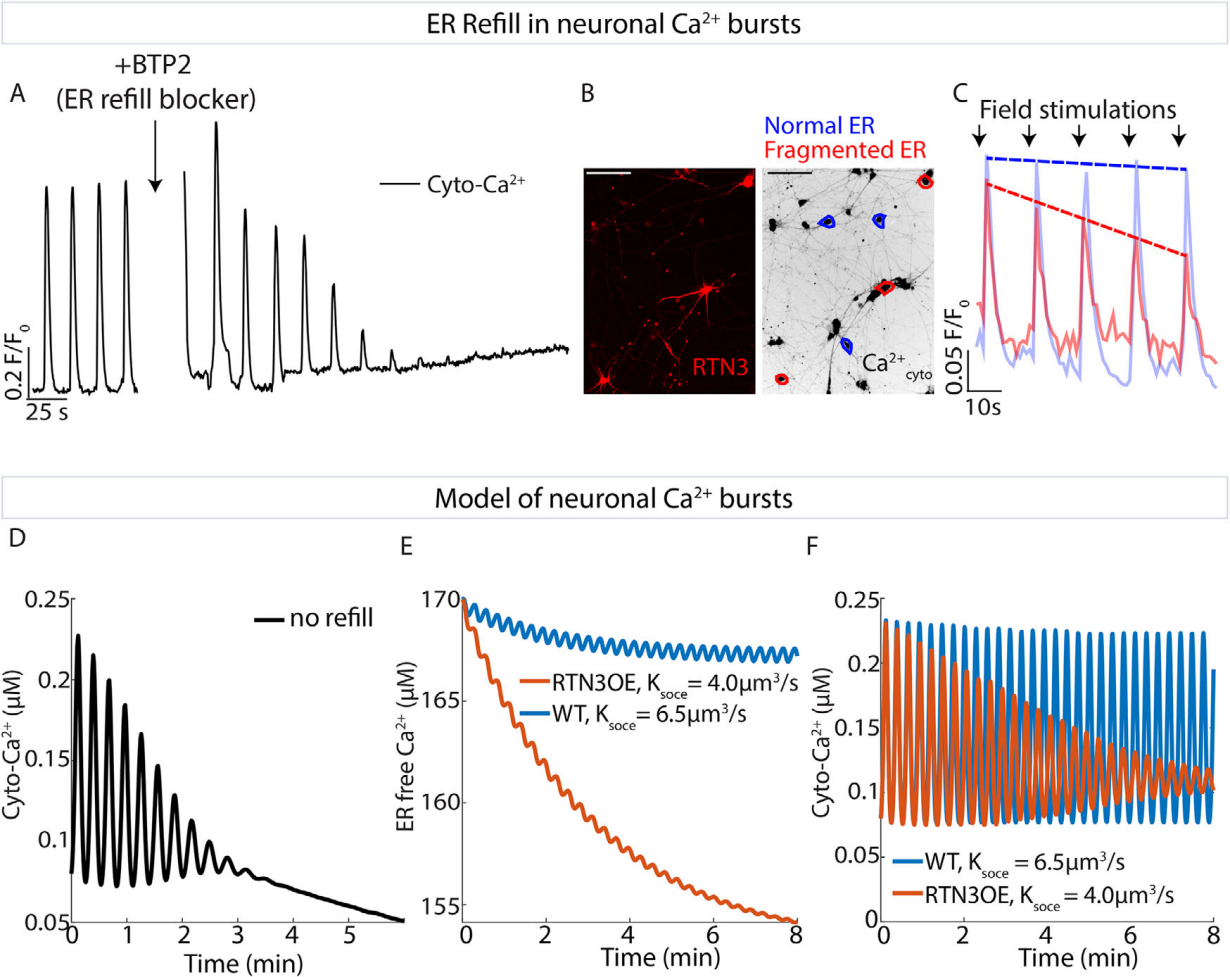
Perturbing ER Refill Hinders Ability to Generate Persistent Bursts. (A) Single‐cell cytosolic Ca^2+^ trace before and after treatment with BTP2 (10 µm, ORAI1 inhibitor). (B) Micrographs of iNeurons with sparse exogenous expression of RTN3a‐Halo labelled with JF646 (left) and stained with the cytosolic Ca^2+^ dye Cal‐520 (right). Blue ROIs: controls (not infected), red ROIS: RTN3aOE cells. Scalebar: 100 µm. (C) Single‐cell cytosolic Ca^2+^ traces of WT (blue) and RTN3aOE (red) iNeurons detected as in (B) after periodic field stimulations (arrows, every 15s). Dotted line represents linear fit of the bursts’ peaks. (D) Spontaneous Ca^2+^ firing in nonlinear dynamic model, with no ER refill (k_soce_ = 0), showing gradual decay of burst magnitude and eventual cessation of release. (E) Unbound Ca^2+^ in the ER lumen, from the spontaneous Ca^2+^ firing model with two different rates of SOCE refill, as estimated from spatial simulations, representing a cell with WT (blue) versus partially fragmented (red) ER. (F) Corresponding cytoplasmic Ca^2+^ levels in the model, showing sustained oscillations with high refill (blue) and gradual dampening with a low refill rate (red).

Since ER fragmentation affects Ca^2+^ refill rates, we hypothesised that cells with disrupted ER shape cannot replenish ER Ca^2^^+^ stores quickly enough to support repetitive global releases. To validate the plausibility of this hypothesis, we first interrogated the extent to which ER refilling limits repetitive firing. To mimic the refill bottleneck without altering ER shape, we again acutely blocked SOCE using the ORAI1 antagonist BTP2. Immediately after treatment, cytosolic Ca^2+^ bursts persisted but their amplitude gradually declined until they were completely abolished (Figure 4A; Video S7). The progressive rundown mirrors the burst decline observed when the ER was slowly depleted with SERCA inhibitors (Figure 1I). These are consistent with the critical contribution of ER Ca^2+^ to fuelling the bursts rather than merely triggering them. The burst abolishment after BTP2‐blockage of ORAI is unlikely to be a consequence of an effect on depolarization, since the MEA‐detectable field potentials persisted in the inhibitor‐treated neurons (Figure S10). Therefore, hindering ER refill alone is sufficient to exhaust the capability of ER to sustain the Ca^2+^ firing activity. Further, repeated, high‐frequency electrical field stimulation of iNeurons with RTN3a‐induced ER fragmentation led to progressively declining cytosolic Ca^2+^ bursts amplitude (Figure 4B,C) — whereas cells with normal ER from the same field of view could sustain repeated Ca^2+^ bursts of consistently high amplitude. The gradual dampening of burst amplitude mirrors the refill inhibition phenotype achieved by BTP2 — while these cells retain normal Ca^2+^ release kinetics (Figure S3). Therefore, we conclude that the slow refilling of fragmented ER causes its failure to sustain the global mode of Ca^2+^ firing.

Our physical model quantitatively determined to what extent limited ER refill rates can account for the observed bursts’ gradual rundown. We implemented a modified version of an archetypal model for Ca^2+^ oscillations [54], incorporating IP3‐receptor mediated Ca^2+^ release from the ER, SERCA‐mediated Ca^2+^ reuptake, cytoplasmic clearance, and refill of the ER via SOCE. This simplified model treats the ER and cytoplasmic Ca^2+^ pools as well mixed, without representing spatial transport effects within each compartment. It uses a Hodgkin–Huxley‐like formalism to describe the time‐dependent activation and inactivation of IP3R in response to cytoplasmic Ca^2+^ levels, enabling the emergence of autonomous Ca^2+^ release pulses with a periodicity of 16 sec, similar to experimental observations (for model and its parameterisation details see Modelling in Methods).

A key parameter in the model is the rate of ER refill through SOCE. If this refill process is completely shut off, then the modelled Ca^2+^ bursts gradually dampen (Figure 4D), as observed upon BTP2 addition (Figure 4A). To represent the overall SOCE refill rate, we use an effective rate constant (k_soce_) extracted from our simulations of intra‐luminal Ca^2+^ transport (Figure S11), which decreases by approximately 40% upon ER fragmentation. This decrease results in a relatively small (<10%) change to the ER luminal Ca^2+^ levels in the model (Figure 4E), potentially explaining the absence of a detectable ER Ca^2+^ drop in our experiments. However, due to the nonlinear nature of the model, this small change can be sufficient to dampen out Ca^2+^ release pulses over a multi‐minute timescale (Figure 4F), matching our experimental observations (Figure 4C). While not quantitatively predictive due to the presence of multiple under‐specified parameters, the Ca^2+^ pulsing model illustrates the plausibility of Ca^2+^ bursts declining due to a moderate decrease in the SOCE refill rate alone.

Thus, ER fragmentation abolishes global Ca^2^^+^ bursts by slowing ER Ca^2^^+^ store refilling, leading to a partial and transient ER Ca^2^^+^ depletion comparable to that induced by CPA, thereby restricting the Ca^2^^+^ release required to sustain cytosolic Ca^2^^+^ bursts mirroring the drug‐induced defect. Collectively, pharmacological inhibitions, spontaneous and stimulated firing assays, ER Ca^2+^ dynamics measurements and mathematical simulations converge on a single conclusion: a continuous, fast‐tunnelling ER network is required in neurons to replenish their Ca^2+^ during global releases; when refilling cannot keep pace with demand because of perturbed ER shape, burst amplitudes gradually decrease until individual activity ceases, thus disrupting the network firing. The high Ca^2+^ demand global sub‐Hz firing is particularly sensitive to diminishing in supply, even when the ms‐scale synaptic transients sourced from extracellular Ca^2+^ pool are unaffected.

**FIGURE 5:**
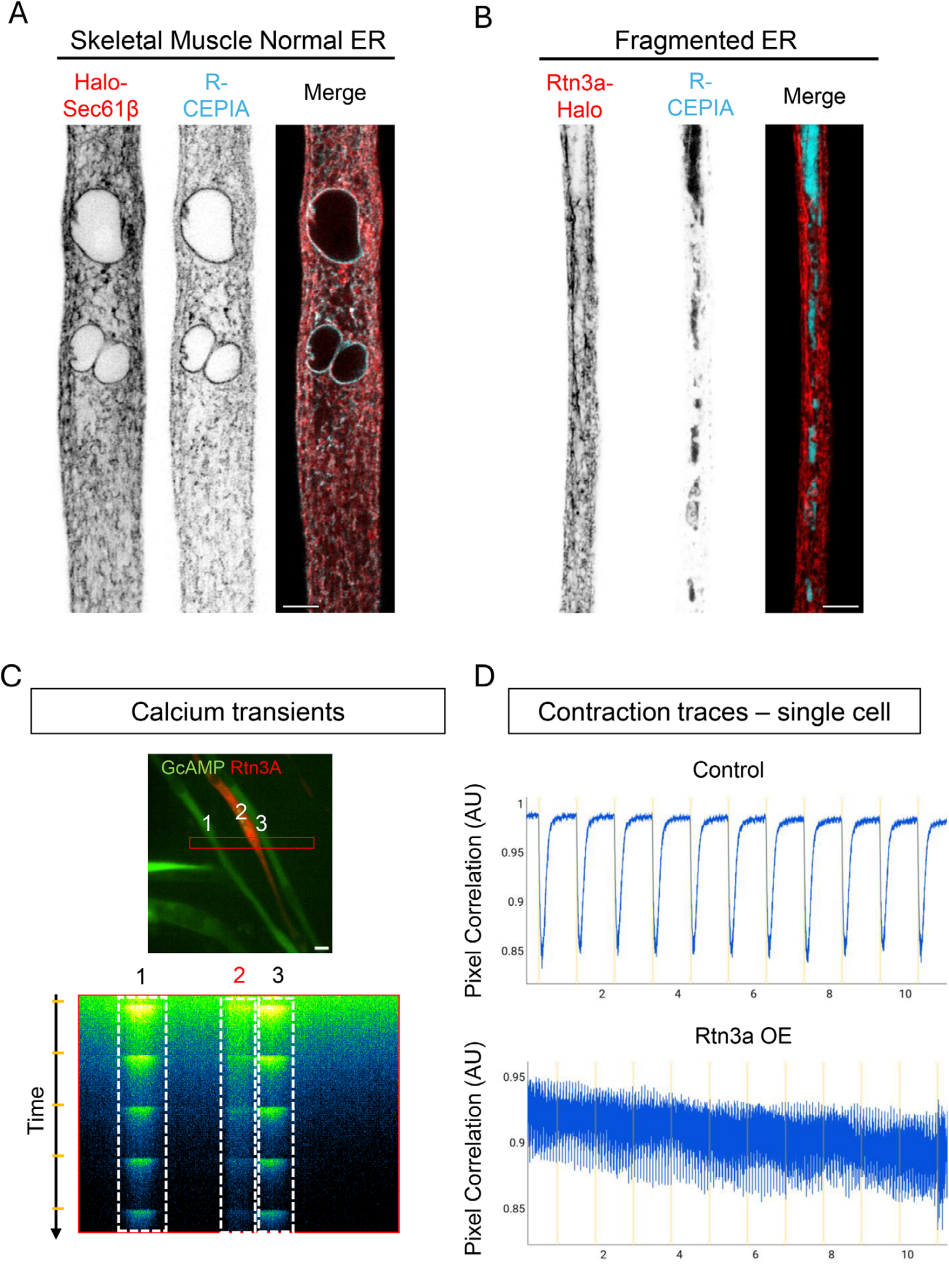
ER With Perturbed Morphology Fails to Support Primary Skeletal Muscle Cell Calcium Handling and Contraction. (A) Micrographs of the ER in primary skeletal muscle cells expressing the ER membrane protein Halo‐Sec61β, labeled with JF646 (red), and the ER luminal marker R‐CEPIA_ER_ (cyan). (B) Micrographs of primary skeletal muscle cells expressing the ER membrane protein RTN3a‐Halo, labeled with JF646 (red), and the ER luminal marker R‐CEPIA_ER_ (cyan). Scale bar, 5 µm. (C) Cytosolic calcium dynamics during electrical stimulation. Top panel: primary skeletal muscle cells expressing the GCaMP calcium reporter (green); cell 2 additionally overexpresses Rtn3a‐Halo (red). Bottom panel: subcellular GCaMP fluorescence kymographs from the boxed region, shown for individual cells. Cells 1 and 3 lack detectable Rtn3a signal and display calcium release and reuptake in response to electrical field stimulation. Cell 2, which overexpresses Rtn3a‐Halo, has residual calcium dynamics. Orange marks indicate electrical pulses. Control cells (positive for Halo‐Sec61β) showed calcium release and uptake in 80% of cells, compared with 27% of Rtn3a‐Halo positive cells. (D) Representative contraction traces from control (Sec61β‐Halo, top) or fragmented ER (Rtn3a overexpression, Rtn3a OE, bottom) skeletal muscle cells during electrical field stimulation. Yellow marks indicate electrical pulses. In control cells (Halo‐Sec61β), 73% of cells exhibited contraction, whereas only 18% of Rtn3a OE cells contracted.

The refill‐speed‐threshold concept—and the newly recognised graded pre‐collapse window— could offer quantitative predictions for other systems with massive Ca^2+^ releases on seconds’ timescale. Skeletal muscles represent one of such systems and depend on the SR – sarcoplasmic reticulum – for Ca^2+^ to initiate contractions [34, 55]. Therefore, we turned to muscle activity readouts to test consequences of ER perturbation on another tissue‐level function. We induced ER fragmentation by RTN3a overexpression in primary mouse skeletal muscle cell cultures (Figure 5A,B). This manipulation abolished the muscle‐cells’ Ca^2+^ transients (Figure 5C) and contractions (Figure 5D), readily inducible by electrical stimulation in normal cells. This supports the contribution of ER morphology to the sustenance of repeated, large scale Ca^2+^ transients potentially extendable to other cell types with similar ER‐dependent Ca^2+^ signalling profiles.

## 3 Discussion

Our results identify a mechanism of ER Ca^2+^ refill‐limited excitability**—**an architectural checkpoint for firing. This constitutes a kinetic bottleneck that caps neuronal network activity: the time required for Ca^2^^+^ to traverse the continuous ER lumen. Perturbing the integrity of tubular ER slows intra‐luminal Ca^2+^ mobility, particularly its tunnelling from the ER plasma membrane contact sites to replenish the store. Although impeded refill is not detrimental for normal Ca^2+^ store maintenance in non‐firing cells (as membrane machinery of Ca^2+^ pumps and channels remain functional in fragmented ER), it is strongly felt when ER is required to support repetitive transient elevations of cytoplasmic Ca^2+^ while firing. During such global release activities, the Ca^2+^‐starved ER struggles to keep up with Ca^2+^ demand and when its contribution fades away, firing stops. Thus, organelle geometry stands alongside ion channels and synaptic receptors as a first‐order determinant of periodic firing, particularly at high amplitude with sub‐Hz frequency.

Modelling, constrained by fluorescence‐recovery measurements, shows burst probability collapsing once the refill rate slows beyond a threshold. Crucially, we observe a progressive decline in burst amplitude before complete failure: partial fragmentation or mild SOCE‐mediated refill inhibition produces mis‐scaled events rather than an abrupt switch‐off. These suggest a possible regime of controlled “luminal starvation” acting as a physiological rheostat: neurons might transiently fragment the ER to dampen network output. Such ER transient fragmentations have been documented in (patho)physiological circumstances with attenuated neuronal activity, particularly in ischemia [56] and after prolonged sensory overstimulation [57]. Further, corticospinal neurons combine millimeter‐scale arbours, distant from the large somatic storage ER reservoirs, with an unfavourable volume‐to‐store ratio, making them sensitive to Ca^2+^ delivery delays. This effect can rationalise why ubiquitous ER‐shaping mutations cripple neurons first and why symptoms often manifest as distal axonopathies. This is consistent with recent observations that hereditary spastic paraplegia (HSP)‐mimicking loss of reticulon in flies reduces terminal Ca^2^^+^ transients and neurotransmitter‐release probability [58, 59]. The effect we described for the seconds‐scale bursts can hold true for shorter time scales and local firing events in a more subtle form. Thus, instead of the all‐or‐nothing scenario for bursts, the effect may translate into weaker synaptic strength or reduced likelihood to fire. Further, complete loss of Atlastin 2/3, another ER morphogen with loss‐of‐function mutations in HSP, also showed retarded refill (Figure S12A–C). However, this was not recapitulated in iNeurons expressing dominant‐negative Atlastin, reflecting a limited ability of the dominant‐negative to affect ER transport and refill in these cells.

This concept should be expected to extend to other systems with similar spatiotemporal scale of Ca^2+^ signalling: consistent with this prediction, ER fragmentation reduced the amplitude of Ca^2+^ spikes and contractility in skeletal muscle. Other systems that may exhibit similar behavior include astrocytes, which also rely on ER stores for their function state‐associated intercellular Ca^2^^+^ waves [33, 36].

By establishing a direct structure‐function relationship for the neuronal ER, we elevate organelle architecture to a quantitative variable in neurophysiology. The principle of refill‐limited excitability suggests that modest geometric tuning—whether pathological or adaptive—can shift networks along a continuum from normal bursting to quiescence. Maintaining ER continuity, therefore, could represent a general strategy for balancing neuronal functionality.

**TABLE 1:** Key resources table.

Reagent or Resource	Source	Identifier
Antibodies		
MAP2 (D5G1) XP Rabbit mAb	Cell Signalling Technology	#8707
Goat anti‐Rabbit IgG (H+L) Highly Cross‐Adsorbed Secondary Antibody, Alexa Fluor 488	Invitrogen	a11034
Bacterial and virus strains		
NEB 5‐alpha Competent E. coli	New England Biolabs	C2987I
Incucyte Neuroburst Orange Lentivirus	Sartorius	4736
AAV9 pAAV.CAG.GCaMP6s.WPRE.SV40	Addgene	100844‐AAV9
Experimental models: Organisms/strains		
C57BL/6 mice, strain code 027	Charles River	N/A
Chemicals, peptides, and recombinant proteins		
Cal‐520, AM	AAT Bioquest	21130
Janelia Fluor 646 HaloTag Ligand	Promega	G8272
Halo‐JFX650	Lavis Lab	N/A
HaloTag Oregon Green Ligand	Promega	G2802
ci‐IP3/PM	Bio‐Techne	6210
Incucyte Cytotox Red Dye	Sartorius	4632
Thapsigargin	Cam Bioscience	10522‐1mg‐CAY
Cyclopiazonic acid (reversible SERCA inhibitor)	Tocris	1235/10
Tetrodotoxin	Abcam	ab120054
CNQX	Gift from Mallucci's lab	N/A
(+)‐MK 801 maleate	Tocris Bioscience	0924
MPEP hydrochloride	Bio‐Techne	1212
LY 367385	Tocris Bioscience	1237
2‐APB	Bio‐Techne	1224/10
Dantrolene, Sodium Salt	Sigma–Aldrich	251680‐100MG
EGTA 0.5 m, pH 7.4, Sterile	bioPLUS	40120370‐2
Histamine	Sigma (Merck)	H7125‐1G
Ionomycin Calcium Salt	PeproTech EC Ltd (BioGems)	5608212‐1
U73122, plc inhibitor	Biotechne (Tocris)	1268
YM 58483	Tocris	3939
Vitronectin (VTN‐N) Recombinant Human Protein, Truncated	Thermo fisher (Gibco)	A14700
Geltrex (LDEV‐free hESC qualified reduced growth factor basement membrane matrix)	Thermo fisher (Gibco)	A1413302
LAMININ FROM ENGELBRETH‐HOLM‐SWARM MURIN	Merk	L2020‐1MG
POLY‐L‐LYSINE SOLUTION, 0.01%, STERILE‐F	Sigma	P4707‐50ML
Cultrex Poly‐L‐Lysine	bio‐techne	3438‐100‐01
StemFlex Medium	Fischer Scientific	15627578
TeSR‐E8 Kit for hESC/hiPSC Maintenance	STEMCELL technologies	5990
Gibco DPBS, no calcium, no magnesum, 500 mL	Thermo Fisher Scientific (Life Technologies)	14190169
ROCK Inhibitor (Y‐27632) 1.0mg	Insight Biotechnology (BD Biosciences)	562822
StemPro Accutase	ThermoFisher Gibco	A1110501
Gibco StemPro Accutase Cell Dissociation Reagent	Fisher Scientific Ltd	11599686
DMEM/F‐12, with GlutaMax, 500 mL	Thermo Fisher	11559726
Penicillin‐Streptomycin	Thermo Scientific (Gibco)	15140122
L‐Glutamine, 100 mL	Thermo Fisher Scientific	25030024
Non‐Essential Amino Acids (NEAA), 100 mL	Gibco	11140035
2‐Mercaptoethanol (50 mm)‐20 mL	Gibco	31350010
N‐2 Supplement (100x) 5mL	Gibco (Thermo Fisher)	17502048
Doxycycline Hydrochloride	fisher scientific	BP2653‐1
Uridine	Merck	U3003‐5G
5‐Fluoro‐2′‐deoxyuridine	Merck	F0503‐100MG
Neurobasal medium	Thermofischer	21103049
BDNF Recombinant Human Protein	Fisher Scientific Ltd	10477253
Recombinant Human NT‐3	Peprotech	450‐03‐10
B27 Supplement	ThermoFisher Scientific	17504044
Opti‐MEM I Reduced Serum Medium	ThermoFisher Scientific	31985062
Trypsin‐EDTA (0.05%), phenol red	ThermoFisher	25300054
DMEM (high glucose, with Glutamine, sodium Puruvate)	sigma	D6429‐500ML
Collagenase type V	Sigma	C9263
Dispase II	Roche	4942078001
Matrigel Growth reduced factor	Corning	354230
Fetal Bovine Serum (FBS)	Eurobio	CVFSVF0001
Chicken Embryo Extract	MP biomedical	2850145
Recombinant rat agrin	R&D systems	550‐AG‐100
Horse Serum	GE Healthcare	11581831
Lipofectamine 2000	ThermoFisher	11668019
Halo‐JF646	Lavis Lab	N/A
Halo‐TMR	Promega	G8251
Critical commercial assays		
PureYield Plasmid Maxiprep System	Promega	A2393
PureYield Plasmid Miniprep System	Promega	A1222
Wizard SV Gel and PCR Clean‐Up System	Promega	A9282
NEBuilder HiFi DNA Assembly Master Mix	New England Biolabs	E2621L
PEI MAX—Transfection Grade Linear Polyethylenimine Hydrochloride (MW 40,000)	Polysciences	24765‐100
GoScript Reverse Transcriptase kit	Promega	A2791
PowerUp SYBR Green Master Mix	Applied Biosystems	A25779
RNeasy Mini Kit	Qiagen	74136
Experimental models: Cell lines		
Human: NGN2 iPSCs	M. Ward (National Institutes of Health) [55]	RRID: CVCL_C7XJ
HEK293T	ATCC	RRID: CVCL_0063
COS‐7	ATCC	RRID: CVCL_0224
Recombinant DNA		
pLV_TRE3G_Halo‐Sec61β	This study	RRID: 254290
pLV_TRE3G_RTN3A‐Halo	This study	RRID: 254291
pLV_TRE3G_Halo‐Neuroserpin_G392E_	This study	RRID: 254292
pLV_CMV_mEmerald‐KDEL	Avezov lab [15]	RRID: 216256
pLV_TRE3G_ ER‐GCaMP3	This study	RRID: 254293
pLV_TRE3G_GCaMP8	This study	RRID: 254294
pLV_TRE3G_mCherry‐KDEL	This study	RRID: 254295
pLV_TRE3G_R‐CEPIA1er	This study	RRID: 254296
ER‐GCaMP6‐150	Addgene	RRID: 86918
pLV_Tre3g_CalcireticulinSS_mTurquoise_D4‐cameleon_ REAch2_KDEL	Avezov lab [15]	RRID: 57132
pLV_Tet3G_GFP‐MAPPER	This study	RRID: 254297
pMD1g/pRRE	Addgene	RRID: 12251
pRSV‐Rev	Addgene	RRID: 12253
pDM2.G	Addgene	RRID: 12259
pLV_TRE3G_ER‐LAR‐GECO1	This study	RRID: 254298
CMV‐ER‐LAR‐GECO1	Addgene	RRID: 61244
pLV_TRE3G_ATL1 K80A‐Halo	This study	RRID: 254299
pCMV R‐CEPIA_ER_	Addgene	RRID: 58216
Software and algorithms		
Fiji (ImageJ v2)	NIH, USA	RRID: SCR_002285
Leica Application Suite X	Leica	RRID: SCR_013673
IncuCyte S3 Live Cell Analysis System	Sartorius	RRID: SCR_023147
Axion Maestro Pro	Axion	N/A
Chamlide electric stimulation chamber	LCI	EC‐B25
A‐M Systems isolated high powered stimulator	A‐M systems	AMS4100
Python v3.10	Python Software Fundation	N/A
Imaris	Oxford Instruments	N/A
ZEN 3.8 blue edition	Zeiss	N/A
MyoPacer	IonOptix	N/A
NIS‐Elements AR software v5.42.05	Nikon Instruments	N/A
IonWizard v7.7.3.180	IonOptix	N/A
Design and Analysis Software v2.5.1	ThermoFisher	N/A
QuantStudio 5 real‐time PCR system	Applied Biosystems	A34322

## 4 Materials and Methods

### 4.1 iPSCs Maintenance and Differentiation Into iNeurons

Materials are listed in Table 1. Human Induced Pluripotent Stem Cells (hiPSCs) with stable integration of NGN2 into a safe‐harbour locus under a doxycycline‐inducible promoter (iNeurons) [37], were cultured in STEM FLEX medium (STEMCELL technologies) on vitronectin (ThermoFisher Gibco)‐coated plates (1:100 dilution, 1 h) and treated with 10 µm Rock Inhibitor (BD biosciences) in the first 24 h after seeding. To induce neuronal differentiation, cells were transferred on Geltrex (ThermoFisher Gibco)‐coated plates (1:100 dilution, 1 h), and kept in STEM FLEX medium supplemented with Rock Inhibitor for 24 h. Then, the STEM FLEX was replaced with DMEM/F12 (Gibco) supplemented with 10 µL/mL N‐2 supplement (Gibco), 2 mm L‐glutamine (Gibco), 10 µL/mL NEAA (Gibco), 50 µm 2‐mercaptoethanol, 10 µL/mL Pen‐Strep (Gibco), and 1 µg/mL doxycycline (Fischer Scientific). This medium was changed daily for two days. On day 3 after induction, the medium was replaced with the maintenance medium Neurobasal (Gibco) supplemented with 20 µL/mL B‐27 (Gibco), 2 mm L‐glutamine (Gibco), 50 µm 2‐mercaptoethanol (Gibco), 10 µL/mL penstrep (Gibco), 1ug/mL doxycycline (Fischer Scientific), 10 ng/mL NT‐3 (Gibco), and 10 ng/mL BDNF (Gibco). At day 4, cells were transferred to different kinds of plates, depending on the analysis modality, coated with poly‐L‐lysine (Bio‐Techne) overnight and laminin (Merk) at a 1:100 dilution for 2 h. The media was supplemented with 3 µm uridine (Sigma–Aldrich), 1 µm 5‐Fluoro‐2′‐deoxyuridine (Sigma–Aldrich), and 10 µm ROCK inhibitor (BD Biosciences) for 24 h after seeding. The maintenance medium was subsequently changed every day for 4 days and every other day after day 8.

### 4.2 MAP2 Immunostaining

Cells were fixed with a pre‐warmed fixation buffer (2% PFA, 2% glutaraldehyde, 100 mm cacodylate (pH 7.4), and 2 mm CaCl_2_) for 30 min at room temperature (RT). After washing with 0.001% Triton in PBS, cell membrane was permeabilized with 0.5% Triton X‐100 in PBS for 5 min. After, neurons were incubated in blocking solution (10% goat serum in PBS) for 1 h. Then, after washing with PBS, samples were incubated overnight with primary antibody (1:500, MAP2 (D5G1) XP Rabbit mAb, Cell Signalling Technology, #8707) in 10% goat serum at 4°C. On the next day, cell cultures were washed and incubated for 1 h with secondary antibodies (1:1000 in PBS, Alexa 488, Invitrogen, goat anti‐rabbit).

### 4.3 Multielectrode Array Field Potentials Recordings

For multielectrode array (MEA) recordings, differentiated neurons were seeded on day 4 on a CytoView MEA 48 plate (Axion Biosystems, M768‐tMEA‐48 W) coated as described above. 10 000 cells resuspended in 10 µL of iN2 and added directly over the electrode region in each well. Once attached, 300 µL of medium was added in each well and changed regularly as described above. Electrical activity was then recorded at ≥ day 21 using the Axis Navigator—Neuronal Activity Module of the Axion Maestro Pro (Axion Biosystems). Single electrode data was then extracted through the Axion Data Export tool and plotted using a custom Python code, while raster plots were generated through the Axion Neural Metric Tool.

For the SOCE inhibition experiment (Figure S10), BTP2 (10 µm) was added to the media, and the recording started immediately after. The network burst frequency from each well was extracted using the Axion Neural Metric Tool, and the mean and std across the wells of each timepoint and condition were calculated and plotted in Python.

### 4.4 Whole‐Network Cyto‐Ca^2+^ Recordings

For whole‐network cytosolic Ca^2+^ recordings, differentiated neurons were seeded on day 4 on a 96‐wells plate (Corning, 3595) at a density of 40 000 cells per well in 150 µL of maintenance medium. Ca^2+^ fluctuations of differentiated iNeurons were assessed using Neuroburst Orange, a lentivirus containing a genetically encoded Ca^2+^ indicator under a synapsin promoter (Sartorius, 4736). At day 7, neurons were transduced with 3 µL of Neuroburst lentiviral solution per well. Infected neurons were recorded at ≥ day 21 using the Neural Activity module of the automatic microscopy imaging system Incucyte (S3neuro, Sartorius). Wells were imaged at 3.33 Hz for 1 min every 20 min using a 4 × objective. The resulting videos were analyzed using the Incucyte software, which calculated active objects counts and mean correlation values of each well. Means and SDs of these values per condition were then calculated and plotted using a custom Python code. The videos and masks of active objects were also extracted to plot the normalised Neuroburst intensity of each active object. Cells were treated by adding the drugs directly into the well, and washed‐out by replacing the medium 3 times. For the RTN3 OE experiment (Figure 2G–I), cells were infected with RTN3 lentiviral particles at Day 8 and maintained in iN2 without DOX until the start of the experiment (day 28), when RTN3 OE was induced by adding DOX.

### 4.5 Lentiviral Particles Production

8 × 10^6^ HEK293FT cells per flask were seeded in T175 flasks (Greiner) and incubated overnight. The next day, we incubated for each flask 2.5 µg of each third‐generation viral packaging plasmids (Addgene #12251, #12253, #12259) and 2.5 µg of the plasmid of interest with 60 µL Polyethylenimine (PEI, Polysciences, 24765‐100) in 1 mL Opti‐MEM (Fisher Scientific) for half an hour and added the solution to the cell culture medium. The media (25 mL per flask) was then replaced every 24 h and collected after 48 and 72 h. The collected media was filtered with a 0.45 µm filter and transferred into 50 mL tubes. 2 mL of 20% sucrose water solution was added at the bottom of each tube which were then centrifugated at 14 000 rcf for 3 h 30 min at 4°C. The formed pellet containing the viral particles was resuspended in 30 µL of PBS per 50 mL of collected media and stored at −80°C.

### 4.6 Single‐Cell Cytosolic/ER Ca^2+^ Recordings in Firing iNeurons

For single‐cell cytosolic Ca^2+^ recordings, differentiated neurons were seeded on day 4 on a µ‐Slide 8‐well polymer bottom dish (IBIDI, 80801) coated as described above, at a density of 100 000 cells per well in 300 µL of maintenance medium. At day 15, wells were transduced with Neuroburst (Sartorius, 6 µL per well) lentiviral particles. At day 28, cells were imaged on a STELARIS8 confocal microscope (Leica, Wetzlar, Germany) with a controlled environment (37°C, 5% CO_2_). ROIs were manually drawn around cells in the FOV in the Leica Stelaris software and their intensity values were extracted and plotted in Python.

For the EGTA experiment (Figure 1H), videos were acquired at 2.3 Hz with a 10× objective, using a 558 nm excitation laser at 5% intensity, an emission detector set at 581–741 nm (50 gain), and the pinhole set at 11.31 AU (600 µm). During the recording (as indicated in the figures), cells were treated by adding EGTA (3 mm) directly into the well.

For the CPA experiment (Figure 1I), ER Ca^2+^ fluctuations were imaged using ER‐GCaMP6‐150 [60], delivered through lentiviral transduction at day 4. Videos were acquired at 2.3 Hz using a 20× objective. The imaging setup was as follows: excitation lasers: 497 nm at 1% intensity, 552 nm at 2% intensity and the emission detectors were set at: 505–545 nm (50 gain) and 599–740 nm (50 gain). The pinhole was set to 5.62 AU (317.9 µm). Cells were treated during the recording (as indicated in the figures) by adding CPA (25 µm) directly into the well.

For recordings after ER‐shape manipulation (Figure 2E,F), the media was replaced by iN2 without dox at day 5. At day 8, cells were transduced with lentivirus containing either RTN3a‐Halo + mEmerald‐KDEL (luminal marker to visualize ER) or Halo‐NS_G392E_. To not affect neuronal maturation, the expression of the constructs under the TRE3G promoter was induced through doxycycline addition (1 µg/mL) after checking that most of the cells in the well had Ca^2+^ bursts. After 1 week from overexpression induction, cells were stained with 1 µm Halo‐JFX650 overnight and washed 3x with media, left to rest for 1 h in the incubator, then washed 1x with medium. Videos acquired at 2.3 Hz using a 63× objective. The imaging setup was as follows: excitation lasers: 558 nm at 5% intensity and 658 nm at 0.1% intensity and the emission detectors were set at: 578–621 nm (50 gain) and 709 – 845 nm (2.5 gain). The pinhole was set to 2.88 AU (275.1 µm). The iNeurons cultures containing NS_G392E_OE cells were imaged at 2.3 Hz using a 20× objective. The imaging setup was as follows: excitation lasers: 558 nm at 4% intensity and 658 nm at 0.5% intensity and the emission detectors were set at: 578 – 621 nm (40 gain) and 691 – 845 nm (2.5 gain). The pinhole was set to 4.33 AU (413.2 µm). Intensity values for each cell in the FOV were extracted from the Leica Stelaris software and plotted in Python. Representative images were smoothed using Gaussian Blur in Fiji (ImageJ).

For the BTP2 experiment (Figure 4A), videos were acquired at 2.7 Hz using a 40× objective. The imaging setup was as follows: excitation laser 558 nm at 1% intensity, and the emission detector was set at 585–838 (50 gain). The pinhole was set to 5 AU (386 µm). During the recording, cells were treated by adding BTP2 (10 µm) directly into the well.

### 4.7 Imaging of ER Shape Manipulation

Differentiation of parental hiPSCs into iNeurons was induced as described above, and cells were seeded on day 4 on a µ‐Slide 8‐well polymer bottom dish (IBIDI, 80801) coated as described above, at a density of 50 000 cells per well in 300 µL of maintenance medium. On the same day, cells were transduced with lentivirus containing either RTN3a‐Halo + D4ER‐Tq (ER luminal marker) or Sec61b + mEmerald‐KDEL (ER luminal marker) or Halo‐NS_G392E_ + ER‐targeted GCaMP3 (ER membrane marker) or ATL_K80A_‐Halo. After 1 week of overexpression, cells were stained with 200 nM Halo Ligand JF646 overnight and washed 3x with media, left to rest for 1 h in the incubator, then washed 1x with medium. Cells were imaged on a STELARIS8 confocal microscope (Leica, Wetzlar, Germany) with a controlled environment (37°C, 5% CO_2_) and imaged using a 63× objective. The imaging setup was as follows. For Sec61b + mEmerald: excitation lasers: 487 at 7% intensity and 646 nm at 7% intensity and the emission detectors were set at 498 – 618 nm (50 gain) and 659 – 834 nm (2.5 gain). The pinhole was set to 0.99 AU (95.1 µm). For RTN3a‐Halo + D4ER‐Tq: excitation lasers: 462 at 5% intensity and 646 nm at 5% intensity. The emission detectors were set at 472–558 and 661–834 nm. The pinhole was set to 0.99 AU (95.1 µm). For NS_G392E_ OE + ER‐targeted GCaMP3: excitation lasers: 488 nm at 5% intensity and 646 nm at 2% intensity. The emission detectors were set at 506–560 and 658–776 nm. The pinhole was set to 0.6 AU (56.9 µm), and a line averaging of 2 was used. For ATL_K80A_‐Halo, excitation lasers: 646 nm at 5% intensity. The emission detector was set at 657–834 nm. The pinhole was set to 0.99 AU (95.1 µm). For the showcase of NSG392E in neurites, excitation lasers: 646 nm at 1% or 10% intensity. The emission detector was set at 655–776 nm. The pinhole was set to 1.01 AU (103.4 µm).

### 4.8 3D Reconstructions and ER‐PM Contacts Quantification

To overcome the low transduction efficiency, MAPPER [51] was transduced in parental hiPSCs, and MAPPER‐positive cells were selected through puromycin. Differentiation of the stable MAPPER overexpressing hiPSCs into iNeurons was induced as described above, and cells were seeded on day 4 on a µ‐Slide 8‐well polymer bottom dish (IBIDI, 80801) coated as described above, at a density of 50 000 cells per well in 300 µL of maintenance medium. On the same day, cells were transduced with lentiviruses containing mCherry‐KDEL and either Halo‐NS_G392E_ or RTN3a‐Halo. After 1 week of overexpression, cells were stained with 100 nm Halo Ligand JF646 overnight and washed 3x with media, left to rest for 1 h in the incubator, then washed 1x with medium. Cells were then imaged on a STELARIS8 confocal microscope (Leica, Wetzlar, Germany) with a controlled environment (37°C, 5% CO_2_). Z‐stacks were acquired using a 63× objective and Δz = 0.304 µm. The imaging setup was as follows: excitation lasers: 488 at 2% intensity, 487 at 1%, 647 nm at 1% intensity, and the emission detectors were set at 496–547, 601–634, and 682–850 nm. The pinhole was set to 0.99 AU (95.1 µm), and a line averaging of 2 was used. Representative 3D projections were generated through the LAS X 3D Visualization module. The raw z‐stacks were analyzed through the IMARIS software, where surfaces for MAPPER positive objects were created. The number of individual MAPPER clusters was then extracted as the number of resulting IMARIS surfaces and plotted in Python.

### 4.9 Sample Processing for Electron Microscopy and SEM Imaging

Samples were fixed in fixative (2% glutaraldehyde/2% formaldehyde in 0.05 M sodium cacodylate buffer pH 7.4, containing 2 mm Ca^2+^ chloride) overnight at 4°C. After washing 5x with 0.05 m sodium cacodylate buffer pH 7.4, samples were osmicated (1% osmium tetroxide, 1.5% potassium ferricyanide, 0.05 m sodium cacodylate buffer pH 7.4) for 3 days at 4°C. After washing 5x in DIW (deionised water), samples were treated with 0.1% (w/v) thiocarbohydrazide/DIW for 20 min at room temperature in the dark. After washing 5x in DIW, samples were osmicated a second time for 1 h at RT (2% osmium tetroxide/DIW). After washing 5x in DIW, samples were block‐stained with uranyl acetate (2% uranyl acetate in 0.05 m maleate buffer pH 5.5) for 3 days at 4°C. Samples were washed 5x in DIW and then dehydrated in a graded series of ethanol (50%/70%/95%/100%/100% dry), and 100% dry acetonitrile, 3x in each for at least 5 min. Samples were infiltrated with a 50/50 mixture of 100% dry acetonitrile/Quetol resin (without BDMA) overnight, followed by 3 days in 100% Quetol (without BDMA). Then, the sample was infiltrated for 5 days in 100% Quetol resin with BDMA, exchanging the resin each day. The Quetol resin mixture is: 12 g Quetol 651, 15.7 g NSA (nonenyl succinic anhydride), 5.7 g MNA (methyl nadic anhydride) and 0.5 g BDMA (benzyldimethylamine; all from TAAB). Samples were placed in embedding moulds and cured at 60°C for 2 days.

Thin‐sections (∼ 100 nm) were cut using an ultramicrotome (Leica Ultracut E) and placed on Melinex coverslips and allowed to air‐dry. The coverslips were mounted on aluminium SEM stubs using conductive carbon tabs and the edges of the coverslips were painted with conductive silver paint. Then, samples were sputter‐coated with 30 nm carbon using a Quorum Q150 T E carbon coater.

Samples were imaged in a Verios 460 scanning electron microscope (FEI/Thermofisher) at 4 keV accelerating voltage and 0.2 nA probe current in backscatter mode using the concentric backscatter detector (CBS) in immersion mode at a working distance of 3.5–4 mm. Stitched maps were acquired using FEI MAPS software using the default stitching profile and 5% image overlap.

ER‐PM contact sites in Figure S7 were measured with ImageJ by manually drawing lines where the membranes were in contact and using the measure function to obtain the length of the drawn segments.

### 4.10 Cytotoxicity Assay

Differentiation of parental hiPSCs into iNeurons was induced as described above, and cells were seeded on day 4 on a 96‐well plate (Corning, 3595) coated as described above, at a density of 20 000 cells per well in 150 µL of maintenance medium. At day 5, the media was replaced by iN2 without dox. At day 8, cells were transduced with lentivirus containing either RTN3a‐Halo, Halo‐NS_G392E_, mEmerald‐KDEL (as a control for ER luminal protein overexpression), or Halo‐Sec61b (as a control for ER membrane protein overexpression). To not affect neuronal maturation, the expression of the constructs under the TRE3G promoter was induced through doxycycline addition (1 µg/mL) at day 21. After 1 week from overexpression induction, cells were stained with 250 nm of Cytotox Red Dye (Sartorius), and Halo was labeled with 1 µm HaloTag Oregon Green Ligand for 1 h and washed 3x with media, left to rest for 1 h in the incubator, then washed 1x with medium. Cells were imaged using an Incucyte S3 (Sartorius) microscope using a 20x objective. The resulting images were analyzed using the Incucyte software, which calculated the area of green + red objects and total green objects per well. The ratio between these two values was then calculated and plotted in Python.

### 4.11 Quantitative Real‐Time PCR

hiPSCs were seeded on 3 wells per condition of a 6‐well dish at ∼ 20% confluency—to reach 100% confluency at day 4 post‐differentiation, when cells stop dividing. iNeurons were differentiated from the hiPSCs as described above. At day 4, Dox was removed from the media. At Day 5, cells were transduced with lentiviruses containing RTN3a‐Halo or Halo‐NSG392E. At Day 21, expression of these constructs was induced by the addition of Dox to the media. At Day 27, 50 nm Halo‐ligand TMR was added to the media. At Day 28, infected and uninfected cells were sorted based on HaloTag‐TMR fluorescence intensity as follows: after one PBS wash, cells were harvested using Trypsin. After centrifugation at 500 g, cells were resuspended in cell sorting buffer consisting of 1x PBS supplemented with 0.5% Bovine Serum Albumin (BSA) to prevent cell aggregation. The sorting tubes containing cell suspension were transferred to a BD FACSMelody Cell Sorter (BD Biosciences) where TMR‐positive cells were sorted from the TMR‐negative. Cells were then centrifugated and frozen at −80 degrees. After thawing, total RNA was extracted using the RNeasy Mini Kit (#74136, Qiagen), followed by reverse transcription performed using the GoScript Reverse Transcriptase kit (#A2791, Promega). Quantitative real‐time PCR on the cDNA was performed on the QuantStudio 5 real‐time PCR system (Applied Biosystems, A34322), using the PowerUp SYBR Green Master Mix (#A25779, Applied Biosystems) for qPCR. Primer sequences (forward and reverse, respectively) were as follows: CHOP (DDIT3), 5′‐GGTATGAGGACCTGCAAGAGGT‐3′ and 5′‐CTTGTGACCTCTGCTGGTTCTG‐3′; GAPDH, 5′‐GTCTCCTCTGACTTCAACAGCG‐3′ and 5′‐ACCACCCTGTTGCTGTAGCCAA‐3′. Relative gene expression levels were calculated using the ΔΔCt method with Design and Analysis Software (ver. 2.5.1, Thermo Fisher Scientific).

### 4.12 XBP1 Splicing Assay

Complementary DNAs (cDNAs) prepared as described in the quantitative RT–PCR section were used for PCR amplification using primers (5’‐AAGAGGAGGCGGAAGCCAAGGGGAATG‐3’ and 5’‐ GAATGCCCAACAGGATATCAGACTCTG‐3’). The thermal cycling conditions consisted of an initial denaturation at 95°C for 1 min, followed by 35 cycles of denaturation at 95°C for 15 s, annealing at 68°C for 30 s, and extension at 72°C for 8 s. PCR products were separated on a 4% agarose gel. The ratio of spliced and unspliced XBP1 products (162 and 188 bp, respectively) was densitometrically quantified using ImageJ.

### 4.13 Free ER Ca^2+^ Concentrations Measurements Through FLIM

Differentiation of parental hiPSCs into iNeurons was induced as described above, and cells were seeded on day 4 on a µ‐Slide 8‐well polymer bottom dish (IBIDI, 80801) coated as described above, at a density of 50 000 cells per well in 300 µL of maintenance medium. On the same day, iNeurons were transduced with the D4ER‐Tq FRET‐FLIM probe [15], and either RTN3‐Halo or Halo‐NS_G392E_. After 1 week of overexpression, cells were stained with 100 nM JF646‐HaloTag ligand overnight. iNeurons were imaged on a confocal microscope (SP8, Leica, Wetzlar, Germany) with a controlled environment (37°C, 5% CO2) using a 63x objective. The following parameters were applied: excitation/emission of 448 (20% power)/460–498 nm, pinhole: 0.5 AU (48 µm), scan speed: 400 Hz and settings were set to reach 5000 photons/pixel. Images were processed using the Leica STELLARIS8 FLIM wizard. ROIs were manually drawn around individual cells and data fitted to a mono‐exponential decay function. Lifetime values were converted to [Ca^2+^]_ER_ using the formula reported in the probe's original paper [42]. The calibration of D4ER‐Tq in iNeurons was performed by inducing maximum ER Ca^2+^ condition with addition of ionomycin (10 µm) and minimal ER Ca^2+^ condition was obtained by adding Thapsigargin (3 µm) to trigger full depletion of the Ca^2+^ stored in the ER.

### 4.14 Total ER Ca^2+^ Load Measurements

Differentiation of parental hiPSCs into iNeurons was induced as described above, and cells were seeded on day 4 on a µ‐Slide 8‐well polymer bottom dish (IBIDI, 80801) coated as described above, at a density of 50 000 cells per well in 300 µL of maintenance medium. On the same day, iNeurons were transduced with RTN3a‐Halo or Halo‐NS_G392E_. After 1 week of overexpression, cells were stained with 100 nm JF646‐HaloTag ligand overnight and then with 5 µm Cal‐520 (AAT Bioquest) for 1 h. iNeurons were imaged on a confocal microscope (SP8, Leica, Wetzlar, Germany) with a controlled environment (37°C, 5% CO_2_). Cells were imaged at 0.39 Hz using a 20× objective. The imaging setup was as follows. Excitation lasers: 499 nm at 2% intensity and 646 nm at 0.01% intensity. The emission detectors were set at: 506–617 nm (10 gain) and 653–795 nm (2.5 gain). The pinhole was set to 9.5 AU (600 µm). During the recordings, Thapsigargin (2 µm) was added directly into the well. ROIs were manually drawn around each cell in the FOV in the Leica Stelaris software, and their intensity values were extracted and plotted against time in Python after smoothing with a Savitzky–Golay filter (order 1, window size 15, mode “mirror”) from Scipy. The area under the curve (AUC) was calculated using the “trapz” function from NumPy on the smoothed signal.

### 4.15 ER Ca^2+^ Release Measurements Through IP3 Photo‐Uncaging

Differentiation of parental hiPSCs into iNeurons was induced as described above, and cells were seeded on day 4 on a µ‐Slide 8‐well polymer bottom dish (IBIDI, 80801) coated as described above, at a density of 50 000 cells per well in 300 µL of maintenance medium. On the same day, iNeurons were transduced with GCaMP8 [57] and RTN3a‐Halo. After 1 week of overexpression, cells were stained with 100 nm JF646‐HaloTag ligand overnight and then treated with 3 µm ci‐IP3/PM (Tocris) for 3 h. iNeurons were imaged on a confocal microscope (STELLARIS 8, Leica, Wetzlar, Germany) with a controlled environment (37°C, 5% CO_2_). Cells were imaged at 1.15 Hz using a 20× objective. The imaging setup was as follows. Excitation lasers: 480 nm at 3.5% intensity and 646 nm at 0.01% intensity. The emission detectors were set at: 489–552 nm (100 gain) and 689–834 nm (10 gain). The pinhole was set to 9.5 AU (600 µm). Following an acquisition of pre‐photo‐uncaging images (3 frames), photo‐uncaging of caged‐IP3 was achieved by illumination (405 nm, 100% laser power) of the whole field of view (775 × 775 µm) for a duration of 130 frames using the Fly mode in the FRAP wizard. ROIs were manually drawn around cells in the FOV in the Leica Stelaris software, and GCaMP8 intensity values were extracted. The intensity at each frame was normalized by the intensity before photo‐uncaging and plotted against time in Python after smoothing with a Savitzky–Golay filter (order 1, window size 20, mode “mirror”) from Scipy. Peaks were detected using the “argmax” function from NumPy on the smoothed signal and the area under the curve (AUC) was calculated using the “trapz” function from NumPy on the smoothed signal.

### 4.16 Luminal Transport Measurements Through FRAP

Differentiation of parental hiPSCs into iNeurons was induced as described above, and cells were seeded on day 4 on a µ‐Slide 8‐well polymer bottom dish (IBIDI, 80801) coated as described above, at a density of 50 000 cells per well in 300 µL of maintenance medium. On the same day, iNeurons were infected with mCherry‐KDEL and RTN3a‐Halo, Halo‐NS_G392E_ or ATL1_K80A_‐Halo. After 1 week of overexpression, cells were stained with 100 nM JF646‐HaloTag ligand overnight. iNeurons were imaged on a confocal microscope (STELLARIS8, Leica, Wetzlar, Germany) with a controlled environment (37°C, 5% CO_2_). Cells were imaged using a 20× objective. For each recording, a circular region of radius ∼ 0.66 µm was selected in the peripheral region of a cell containing vesicular ER or a normal ER network. The imaging setup was as follows: excitation/emission of 587 nm (0.2% power)/597‐623, the pinhole was set to 47.8 µm (0.5 AU). A 646 nm laser was used to identify the transduced cells but not recorded during FRAP. The evolution of the fluorescence signal of mCherry‐KDEL was recorded at 3.7 Hz. Following an acquisition of pre‐photo‐bleaching images (5 frames), photobleaching was performed inside the circular region for 2 frames (0.54 s) by setting the 587 nm laser power to 100%. The recordings were processed in ImageJ [58, 59] with the FRAP profiler v2 plugin (Hardin lab) with the following setup: mono‐exponential fit, ROI1: bleaching ROI, ROI2: entire image. The resulting t‐halves were plotted in Python.

### 4.17 ER Ca^2+^ Refill Measurements

Differentiation of parental hiPSCs into iNeurons was induced as described above, and cells were seeded on day 4 on a µ‐Slide 8‐well polymer bottom dish (IBIDI, 80801) coated as described above, at a density of 50 000 cells per well in 300 µL of maintenance medium. On the same day, iNeurons were infected [57] with RTN3a‐Halo or Halo‐NS_G392E_ or ATL1_K80A_‐Halo. iNeurons were further transduced with ER Ca^2+^ sensors, respectively ER‐GCaMP6‐150^60^ for RTN3 and NS_G392E_ OE, while LAR‐GECO [61] was used for ATL1_K80A_ for its lower KD, to increase the sensitivity to potential small changes at the start of the ER refill process – as previously, refill defects in ATLKO cos cells were only detected using LAR GECO (Figure S10). After 1 week of overexpression, cells were stained with 100 nm JF646‐HaloTag ligand overnight. iNeurons were imaged on a confocal microscope (STELLARIS8, Leica, Wetzlar, Germany) with a controlled environment (37°C, 5% CO_2_). The iNeurons cultures containing RTN3 OE cells were imaged at 1.13 Hz using a 40× objective. The imaging setup was as follows: excitation lasers: 496 nm at 1.5% intensity and 661 nm at 0.01% intensity and the emission detectors were set at: 510 – 554 nm (50 gain) and 713 – 834 nm (50 gain). The pinhole was set to 5 AU (386 µm). The iNeurons cultures containing NS_G392E_ OE cells were imaged at 0.10 Hz using a 20× objective. The imaging setup was as follows: excitation lasers: 496 nm at 3% intensity and 646 nm at 0.01% intensity, and the emission detectors were set at: 514–620 nm (50 gain) and 654–805 nm (50 gain). The pinhole was set to 5.1 AU (288.9 µm). The iNeurons cultures containing ATL1_K80A_ cells were imaged at 0.10 Hz using a 20× objective. The imaging setup was as follows: excitation lasers: 561 nm at 1.5% intensity and 660 nm at 0.01% intensity and the emission detectors were set at: 569–599 nm (80 gain) and 702–834 nm (2.5 gain). The pinhole was set to 5.1 AU (288.9 µm). ROIs were manually drawn around cells in the FOV in the Leica Stelaris software, and GCaMP6‐150 intensity values were extracted. Values were plotted in Python after normalization $f^{\prime }=\frac{f-f_{min}}{f_{max}-f_{min}}$. The sigmoid function $y=1-\frac{1}{1+e^{k}}$ was fitted to this normalised trace using the Curve Fit function from Scipy, where k is the logistic growth rate, and x_0_ is the x value of the function's midpoint. X_0_ (time to half refill) was reported as a measure of refill speed.

### 4.18 SOCE Assessment

Differentiation of parental hiPSCs into iNeurons was induced as described above, and cells were seeded on day 4 on a µ‐Slide 8‐well polymer bottom dish (IBIDI, 80801) coated as described above, at a density of 100 000 cells per well in 300 µL of maintenance medium. On the same day, iNeurons were infected with RTN3a‐Halo or Halo‐NS_G392E_. After 1 week of overexpression, cells were stained with 100 nm JF646‐HaloTag ligand overnight. On the day of the experiment, cells were incubated with 4 µm Cal‐520 (AAT Bioquest) for 1 h. iNeurons were imaged on a confocal microscope (STELLARIS8, Leica, Wetzlar, Germany) with a controlled environment (37°C, 5% CO_2_). Cells were imaged at 0.78 Hz using a 20× objective. The imaging setup was as follows: excitation lasers: 520 nm at 2% intensity and 646 nm at 0.05% intensity, and the emission detectors were set at: 527–620 nm (80 gain) and 655–776 nm (20 gain). The pinhole was set to 3 AU (183.3 µm). Cells were incubated for 5 min with 2.5 mm EGTA to chelate extracellular Ca^2+^ before imaging. Then, Thapsigargin (2 µm) was added to the media. This caused ER Ca^2+^ to leak to the cytosol, giving a first cytolic spike. When cytosolic Ca^2+^ returned to baseline (i.e., most of the ER was emptied from Ca^2+^), the media was replaced with Ca^2+^‐containing media. The resulting cytosolic spike represents SOCE [53]. ROIs were manually drawn around cells in the FOV in the Leica Stelaris software, and Cal‐520 intensity values were extracted. Intensities were plotted against time in Python, and the SOCE ascent trace was smoothed with a Savitzky–Golay filter (order 1, window size 15, mode “mirror”) from Scipy and normalized as $f^{\prime }=\frac{f-f_{min}}{f_{max}-f_{min}}$. The sigmoid function $y=1-\frac{1}{1+e^{k}}$ was fitted to this normalised trace using the Curve Fit function from Scipy, where k is the logistic growth rate, which was reported as a measure of refill speed.

### 4.19 Cyto‐Ca^2+^ Recordings After Refill Inhibition Through BTP2

Differentiation of parental hiPSCs into iNeurons was induced as described above, and cells were seeded on day 4 on a µ‐Slide 8‐well polymer bottom dish (IBIDI, 80801) coated as described above, at a density of 100 000 cells per well in 300 µL of maintenance medium. Cytosolic Ca^+^ fluctuations were imaged using Neuroburst Orange (Sartorius, 6 µL per well), delivered through lentiviral transduction at day 15. At day 28, cells were imaged on a STELARIS8 confocal microscope (Leica, Wetzlar, Germany) with a controlled environment (37°C, 5% CO_2_). Cells were imaged at 2.7 Hz using a 40× objective. The imaging setup was as follows: excitation laser 558 nm at 1% intensity, and the emission detector was set at 585–838 (50 gain). The pinhole was set to 5 AU (386 µm). During the recording, cells were treated by adding BTP2 (10 µm) directly into the well. Intensity values for each cell in the FOV were extracted from the Leica Stelaris software and plotted in Python.

### 4.20 Field Stimulation

Differentiation of parental hiPSCs into iNeurons was induced as described above, and cells were seeded on day 4 on 25 mm glass coverslips coated as described above, at a density of 1 × 10^6^ cells per well in 2 mL of maintenance medium. At day 5, the media was replaced by iN2 without dox. At day 8, cells were transduced with RTN3a‐Halo + mCherry‐KDEL (luminal marker to visualize ER vesicles). To not affect neuronal maturation, the expression of the RTN3‐Halo under the TREG promoter was induced through doxycycline addition (1 µg/mL) after day 20. After 1 week from overexpression induction, iNeurons were stained with 5 µm Cal‐520 and 200 nm JF646 Halo‐ligand for 1 h. Coverslips were mounted into an EC‐B25 stimulation chamber (Chamlide) and imaged using a Nikon Ti2 widefield microscope using a custom stage insert and 20x objective at 37°C and 5% carbon dioxide. Stimulation was performed using an AMS4100 stimulator (A‐M Systems) and dedicated software. The field of view was adjusted to encompass cells with and without overexpression of RTN3. Repetitive stimulation experiments were conducted using a biphasic 1 ms waveform and a voltage setting of 16 V. iNeurons were stimulated with bursts of 50 Hz stimulation, each lasting 0.2 s, at 15‐second intervals.

### 4.21 Cyto and ER Ca^2+^ Recordings in COS7 Cells During EGTA + Histamine Treatment

COS‐7 cells stably expressing the Tet‐On element were transduced with GCaMP8 [62] and R‐CEPIA_ER_ [63]. Cells were then seeded on a 4‐well dish (Greiner Bio‐One, 627975) and expression of the Ca^2+^ sensors was induced by adding Dox (1 µg/mL) for 3 days. Cells were imaged on a STELARIS8 confocal microscope (Leica, Wetzlar, Germany) with a controlled environment (37°C, 5% CO_2_). Cells were imaged at 0.78 Hz using a 20× objective. The imaging setup was as follows: excitation lasers: 480 nm at 1% intensity, 562 nm at 2% intensity, and the emission detectors were set at: 493–550 nm (50 gain) and 611–834 nm (50 gain). The pinhole was set to 10.47 AU (600 µm). Cells were pre‐incubated with EGTA (3 mm) for 5 min, and Histamine (100 µm) was added during the recording (as indicated in the figure). ROIs were manually drawn around cells in the FOV in the Leica Stelaris software, and intensity values were extracted and plotted in Python.

### 4.22 ER Ca^2+^ Refill in COS7 Cells After Histamine‐Induced Release

For global ER Ca^2+^ release induced by Histamine, cells were transfected by electroporation using the Neon Transfection System (Invitrogen) to express ER‐LAR‐GECO1 (together with RTN3E‐Halo for RTN3 OE cell line). Cells were imaged 48–72 h post‐transfection on a confocal microscope (STELLARIS8, Leica, Wetzlar, Germany) with environmental control of the stage (37°C, 5% CO2). Cells were imaged at 2 Hz using a 20× objective. The imaging parameters were: Excitation / emission‐GCaMP3: 480 / 485−550 nm, ER‐LAR‐GECO1: 550 / 600−670 nm, JF646: 646 / 670−750 nm. Cells were incubated with BAPTA‐AM (100 µm, 15 min), and histamine (100 µm) was added during recording (as indicated in the figure). Images were acquired for 5 min post‐treatment. Image analysis was done using Fiji, and a custom code written in Python. Cells were manually segmented, and fluorescence intensity time series within cell outlines were extracted using Fiji and normalized to the intensity measured at t_0_. ER Ca^2+^ drops were automatically detected in intensity traces using Python's function find peak. Intensity traces were smoothed using a moving average with an 8‐frame window. Refill rates were obtained by fitting a sigmoid curve to the smoothed intensity traces, from the lowest value to an end point defined by the length of the fit parameter during analysis: $y=\frac{L}{1+e^{-k}}$, where L is the amplitude of the curve fit and k the logistic growth rate, with k reported as the refill rate.

## 5 Experiments in Primary Skeletal Muscle Cells

### 5.1 Primary Skeletal Muscle Cell Culture

All animal experiments were conducted with approval from the institutional ethics committee and in accordance with the National Research Council Guide for the Care and Use of Laboratory Animals. Primary skeletal muscle cells were generated following an established protocol [64] using primary myoblasts isolated from mixed‐gender C57BL/6 mice aged 5–7 days. Tibialis anterior, extensor digitorum longus, gastrocnemius, and quadriceps muscles were excised and maintained in ice‐cold PBS. The tissue was finely minced with surgical scissors and enzymatically dissociated for 90 min at 37°C under agitation using collagenase type V (Sigma) and dispase II (Roche) prepared in DPBS. Enzymatic activity was quenched by adding IMDM supplemented with GlutaMAX, 10% (v/v) fetal bovine serum (FBS), and 1% (v/v) penicillin–streptomycin. The resulting cell suspension was centrifuged at 75 × g for 5 min to eliminate tissue debris, followed by a second centrifugation at 350 × g for 5 min. Cells were then resuspended in the same medium and pre‐plated for 4 h to allow preferential adhesion of fibroblasts. Non‐adherent cells were collected, centrifuged again at 350 × g for 5 min, and seeded at a density of 2.2 × 10^5^ cells/mL in 35‐mm FluoroDishes (world precision instruments) coated with 1% (v/v) Matrigel in growth medium (IMDM with GlutaMAX, 20% (v/v) FBS, 1% (v/v) Chicken Embryo Extract (CEE) and 1% (v/v) penicillin–streptomycin). After three days, differentiation was induced by switching to IMDM with GlutaMAX supplemented with 10% (v/v) horse serum and 100 ng/mL recombinant agrin. On the following day, an ice‐cold overlay of 50% (v/v) Matrigel prepared in differentiation medium was applied, and fresh medium was added. Experiments were carried out 3–7 days after the onset of differentiation.

### 5.2 Imaging of ER Shape Manipulation

To manipulate ER shape in primary skeletal muscle cells, Rtn3A‐Halotag or Sec61β‐Halo (control, provided by Jennifer Lippincott–Schwartz lab), plasmids were co‐transfected with the luminal marker R‐CEPIA_ER_^63^plasmid to primary skeletal muscle cells. Transfections were performed three days after plating (DIV3) with 1 µg of DNA per ibidi‐35mm‐dish using Lipofectamine 2000 (Thermo), according to the manufacturer's protocol. To visualize Halotag, cells were stained with 200 nm Halo Ligand JF646 for 15 min and washed 3x with media. To visualize ER shape, DIV7 cells were imaged by Airyscan using a Zeiss LSM980 Airyscan2 microscope, equipped with a 63x Plan‐Apochromat DIC oil objective (NA 1.4) at 37°C with 5% of CO_2_ using the ZEN 3.8 blue edition. Airyscan reconstruction was performed using automatic filter settings.

### 5.3 Primary Skeletal Muscle Single‐Cell Contraction and Calcium Dynamics

Mature primary skeletal muscle cells were imaged on a Nikon Eclipse Ti microscope. Throughout imaging and stimulation, cells were maintained at 37°C in a humidified atmosphere containing 5% CO_2_ using an Okolab stage‐top incubator. Excitation–contraction coupling was evoked by electrical field stimulation delivered via the MyoPacer system (IonOptix), using 10 V pulses at 1 Hz with a pulse duration of 20 ms.

Cytosolic calcium dynamics during stimulation were assessed by infecting cells at differentiation day 3–4 with AAV9‐pAAV.CAG.GCaMP6s.WPRE.SV40 (Addgene viral prep #100844‐AAV9; plasmid provided by Douglas Kim and the GENIE Project [65] at a concentration of 1 µL/mL. Following overnight infection, cultures were switched back to differentiation medium (IMDM with GlutaMAX supplemented with 10% (v/v) horse serum and 100 ng/mL recombinant agrin). At differentiation day 7, GCaMP fluorescence responses to electrical field stimulation were acquired using a 60× Plan Apo DM Ph3 objective (NA 1.4) and an Andor Sona CSC‐00324 camera, at a frame interval of 20 ms, controlled by NIS‐Elements AR software (version 5.42.05). A binning of 2 × 2 was used.

For analysis of single‐cell contraction, electrical field stimulation was applied as described above. Contractile activity of individual cells was recorded using a MyoCam‐S3 camera (IonOptix) mounted on a 40 × CFI Plan Apochromat Lambda D objective (NA 0.95), with data acquisition performed using IonWizard software (version 7.7.3.180). Contraction dynamics were derived from changes in pixel correlation calculated by the CytoMotion module (IonOptix), relative to a resting reference frame acquired prior to stimulation. Pixel correlation traces (arbitrary units) were generated using Cytosolver Cloud software (IonOptix). Primary muscle cells were subsequently categorized based on their response to stimulation as non‐contracting or contracting.

### 5.4 Statistical Tests

All statistics were computed in Python using the ttest_ind function from Scipy. All values are reported as average ± STD except when indicated otherwise.

## 6 Modelling

### 6.1 Transport Through Network of Spheres

For a well‐connected tubular lattice, the effective 3D diffusivity of particles embedded in the network is given by $D_{\mathrm{eff},\;\mathrm{tubes}}=\frac{1}{3}\;D$, where *D* is the diffusivity inside the lumen. The factor of 1/3 arises because the particles are only able to move along one dimension at a time when diffusing through the tubules.

To derive the effective diffusivity for a network of large vesicles, we first consider the steady‐state rate of transport for diffusive particles moving between two spheres of Radius *R* and volume *V*, connected by a tube of radius *r* and length *l*. The steady‐state assumption is valid when the spatial concentration profile in the tube equilibrates much faster than the concentration difference between the two spheres, as is the case for very narrow tubules (*r* ≪ *R*). In this situation, we can define the steady‐state concentration in the two spheres as *C*_1_and *C*_2_, and the concentrations at the entrance and exit of the tube as *C*_1*t*_ and *C*_2*t*_.

Using standard results for exit through a narrow pore [66, 67], we can express the current out of the first sphere and into the second sphere as *I*_pore_ = 4*Dr* (*C*_1_ − *C*_1*t*_) = 4*Dr*(*C*_2*t*_ − *C*_2_). The current through the tube is given by Fick's law [66] as *I_tube_* = π*r* ^2^ *D*(*C*_1*t*_ − *C*_2*t*_)/*l*. Since all currents are equal at steady state, we can solve for the concentration in the tube to yield the overall current $I=\frac{2rD}{1+2l/\pi r}\;\left(C_{1}-C_{2}\right).$ Note that this is equivalent to adding three diffusive resistances in series corresponding to the transitions from the first sphere to the tube, across the tube, and from the tube to the second sphere [68]. From the steady‐state current, we can estimate the mean first passage time to transition from one sphere to another, as the inverse of the current when the first sphere contains a single particle and the second sphere is empty *C*_1_ = 1/*V*, *C*_2_ = 0, giving

(1) $$\tau =\frac{1}{I}=\frac{Vl}{D\pi r^{2}}+\frac{V}{2rD}$$

The timescale to refill an empty sphere connected through tubules to *n* neighboring spheres is simply τ/*n*. For a lattice of vesicles with radius *R* connected by tubules of length *l*, we can approximate the particle motion as a series of discrete hops (transitions between spheres), each requiring time τ/*n* and each shifting the particle by a distance 2*R* + *l* along a single dimension. The resulting effective diffusivity is then

(2) $$D_{\mathrm{eff},\mathrm{vesc}}=\frac{1}{3}\;\frac{{\left(2R+l\right)}^{2}n}{2\tau }\;\overset{r\ll I,R}{\to }\;\frac{n{\left(2R+l\right)}^{2}Dr^{2}}{8R^{3}l}\;\overset{l\ll I,R}{\to }\;\frac{nDr^{2}}{2Rl}$$

Here, the first limit corresponds to *r* ≪ *l*, *R*, and the second limit additionally assumes *l* ≪ *R*. The final expression emphasizes the strong slowdown of spatial transport when large vesicular structures are connected by very narrow tubules.

For the vesicular lattice considered here (with *r* = 0.018 µm*, R* = 0.6 µm*, l* = 0.3 µm*, n* = 6), the ratio of effective diffusivities is *D*_eff, vesc_/*D*_eff, tubes_ ≈ 0.023. This estimate implies that diffusive particles will move much more slowly across an unbounded network of vesicles connected by narrow tubules (as for the RTN3OE ER structure) than they will in a purely tubular network (as in the WT ER structure). The vesicles serve as traps for diffusive particles, which must find narrow tubule entrances to escape. Larger spheres are expected to yield slower transport, as observed experimentally for the comparison of RTN3OE and NS architectures.

### 6.2 Simulations of Network Ca^2+^ Refill

To specifically explore the effect of Ca^2+^ transport and network structure on the refill process, we made use of a spatially resolved model that eschews all other aspects of Ca^2+^ dynamics. This model was described in our previous work [15]; source code is available at https://github.com/lenafabr/networkSpreadFVM. This model treats the WT ER as a 3D network of narrow tubules, enclosed within a spherical cell, with a finite number of contacts to the plasma membrane at the cell periphery. At these contacts, SOCE is assumed to be rapid enough to maintain a fixed free Ca^2+^ concentration of 0.5 mm within the ER lumen. Diffusive transport of both free and bound Ca^2+^ ions then determines the typical rate of refilling for the entire network. The default number of PM contact sites (in Figure 3G,H) is set to 40.

### 6.3 Network Construction

The 3D tubular network is constructed by starting with a diamond lattice and randomly shifting one edge at each node to leave junctions of degree 3. The average edge length is 1 µm. The network is cropped to lie within a spherical shell of outer radius R_out_ = 10 µm (representing the cell boundary) and inner radius 5 µm (representing the nucleus). The network is then connected to a well‐mixed perinuclear reservoir with a volume of 189 µm^3^, corresponding to 10 sheets of thickness 0.06 µm surrounding a nucleus of radius 5 µm. Figure S7 shows the effect of increasing the domain size R_out_ on the overall network refill.

For the partially fragmented ER structure, we begin with a hexagonally close‐packed lattice of nodes and randomly disconnect edges to reach an average degree 6 at each node. The individual nodes then represent well‐mixed spherical vesicles. We set the vesicle radius to 0.6 µm for the RTN3OE structure and to 0.5 µm for the NS structure, based on measurements of the ER bubbles observed in 2D images (as in Figure 2B). Each bubble was connected to its (on average) 6 neighbours by a tubule of length 0.3 µm. The tubule radius was selected based on the observed rate of fluorescent protein recovery following photobleaching of an individual bubble (Figure 3C). Using the analytic approximation for the rate of transfer between individual spheres (Equation (1)), and assuming a connectivity of *n* = 6, tubules of radius 0.018 µm result in an average refill time of τ/*n* = 16 sec and τ/*n* = 9 sec for spheres of radius 0.6, 0.5 µm, respectively. As for the purely tubular case, the vesicular network was connected by short tubules to a perinuclear reservoir.

For each network structure, a random subset of peripheral nodes is selected to represent plasma membrane (PM) contact sites. We assume rapid local refill through SOCE at these sites, fixing the free Ca^2+^ concentration to 0.5 mm at the PM contacts. Although iNeurons exhibit extensive narrow projections, such projections are expected to contain their own frequent PM contact sites [52] and thus should refill quickly compared to the bulk ER in the soma. Figure S13 demonstrates that inclusion of a long narrow projection has little effect on the overall filling rate.

The rate of ER refilling is determined by a combination of total network volume, rate of SOCE at ER‐PM contact sites, and transport dynamics through the network. Our base model assumes local Ca^2+^ entry through SOCE is arbitrarily fast, and the perinuclear volume is adjusted to give a refill half‐time of 200 sec, similar to observations in vivo. An alternate model incorporates limited entry at the contact sites, with current I_SOCE_ = P(U_ext_ – U_PM_), with U_ext,_ U_PM_ being the free Ca^2+^ in the external medium and in the local ER lumen at the contact site. Low values of P correspond to slower refill, and the system becomes more sensitive to further decreases in local SOCE rates. Figure S9 provides a lower‐bound estimate for P and demonstrates that even in this limit, overall network refill is more sensitive to changes in ER morphology than to moderate decreases in the local SOCE rate.

### 6.4 Buffer Binding

In both WT and fragmented networks, the intra‐luminal space is assumed to contain buffer sites with binding strength *K_D_* = 0.2 mm. For the WT network, the total concentration of buffer sites is set to *S* = 2.7 mm, such that the total Ca^2+^ concentration in the filled lumen is five‐fold higher than the free Ca^2+^. While the vesicular network has a larger total volume than the tubular one, we assume that the total number of buffer proteins remains the same across both morphologies. Consequently, the buffer concentration was scaled down in proportion to the volume ratio between the two.

Buffer proteins were taken to have a diffusivity of *D_b_* = 3 µm^2^ */*s [16], while free Ca^2+^ was taken to diffuse ten‐fold faster at *D_c_* = 30 *µ*m^2^ */*s. The concentration field of free Ca^2+^ is propagated forward using a finite volume approach, under an assumption of rapidly equilibrated binding, as described in prior work [15].

### 6.5 Extracting Refill Rate Estimate

To estimate a single rate constant for refill from the simulations, we track both ⟨*U*(*t*)⟩ (the free Ca^2+^ concentration averaged across the entire ER lumen) and the current *I*(*t*) of Ca^2+^ ions entering through the PM contact sites. The effective refill rate constant is then computed as *k*_SOCE_ = *I*(*t*)/(*c_cs_* − 〈*U*(*t*)〉), where *c*_cs_ is the fixed free Ca^2+^ concentration at the PM contacts. Following an initial rapid peripheral filling phase, *k*_SOCE_(*t*) reached a plateau during a quasi‐steady‐state where peripheral Ca^2+^ became saturated, but the reservoir remained only partially filled.

### 6.6 Mathematical Model for Ca^2+^ Oscillations

To describe the dynamics of periodic Ca^2+^ pulses, we implemented an aspatial model that encompasses the interchange of ions between two well‐mixed pools: the ER lumen and the cytoplasm. Our approach modifies a classic simplified model developed by Li and Rinzel [54], which incorporates dynamic Ca^2+^‐dependent activation and inactivation of IP3 receptors, as well as SERCA pumping and clearance from the cytoplasm. This model is extended here to explicitly include Ca^2+^ buffers in the ER lumen and refilling of the ER through SOCE.

The model tracks the dynamics of *u*_ER_ (concentration of free Ca^2+^ in the ER lumen) and *c*_cyto_ (cytoplasmic Ca^2+^ concentration). The total luminal Ca^2+^ is set to *c_ER_* = *u_ER_* [1 + *S*/(*u_ER_* + *K_D_*)]. Cytoplasmic buffering is not included. The concentrations evolve based on Ca^2+^ fluxes according to

(3) $$\frac{dc_{\mathrm{cyto}}}{dt}=\frac{1}{V_{\mathrm{cyto}}}\;\left(J_{\mathrm{IP}3R}+J_{\mathrm{leak}}-J_{\mathrm{pump}}-J_{\mathrm{out}}\right)$$

(4) $$\frac{dc_{\mathrm{ER}}}{dt}=\frac{1}{V_{\mathrm{ER}}}\;\left(-J_{\mathrm{IP}3R}-J_{\mathrm{leak}}+J_{\mathrm{pump}}+J_{\mathrm{SOCE}}\right)$$

where the individual fluxes are defined as follows:

(5) $$J_{\mathrm{IP}3R}=v_{1}\;m_{\infty }^{3}h^{3}\left(u_{\mathrm{ER}}-c_{\mathrm{cyto}}\right)$$

(6) $$J_{\mathrm{leak}}=v_{2}\left(u_{\mathrm{ER}}-c_{\mathrm{cyto}}\right)$$

(7) $$J_{\mathrm{pump}}=v_{3}\;\frac{c_{\mathrm{cyto}}^{2}}{k_{\mathrm{ER}}^{2}+c_{\mathrm{cyto}}^{2}}$$

(8) $$J_{out}=v_{4}\;\frac{c_{\mathrm{cyto}}^{2}}{k_{\mathrm{PL}}^{2}+c_{\mathrm{cyto}}^{2}}$$

(9) $$J_{SOCE}=k_{\mathrm{SOCE}}\;\left(c_{CS}-u_{ER}\right)$$

*J*_IP3R_ defines the flux through IP3‐gated Ca^2+^‐responsive Ca^2+^ channels. Each IP3 receptor subunit is assumed to contain one regulatory site for IP3 binding, one for Ca^2+^ activation, and one for Ca^2+^ inhibition. The probability of the channel being open depends on the state of each of the regulatory sites. The IP3 binding site and the Ca^2+^ activation site are assumed to equilibrate instantaneously to the steady state level:

(10) $$m_{\infty }=\left(\frac{\left[IP_{3}\right]}{\left[IP_{3}\right]+d_{ip3}}\right)\;\left(\frac{c_{\mathrm{cyto}}}{c_{\mathrm{cyto}}+d_{act}}\right)$$

The inhibitory site responds to Ca^2+^ more slowly, and its state is defined by the Hodgkin–Huxley‐like gating variable *h*(*t*), which evolves according to

(11) $$\frac{dh}{dt}=\frac{h_{\infty }-h}{\tau_{h}}\;$$

with steady‐state *h*_∞_ and time constant τ_*h*_:

(12) $$h_{\infty }=\frac{Q_{2}}{Q_{2}+c_{\mathrm{cyto}}}\;,\;\;\tau_{h}=\frac{1}{a\left(Q_{2}+c_{\mathrm{cyto}}\right)}\;,\;\;Q_{2}=d_{2}\;\frac{\left[IP_{3}\right]+d_{1}}{\left[IP_{3}\right]+d_{3}}$$

The cubic dependence of the regulatory sites was previously extracted from experimental fits [54].

The flux *J*_leak_ accounts for passive leakage of Ca^2+^ from the ER to the cytoplasm, set to 0 in our version of the model. *J*_pump_ denotes active reuptake of Ca^2+^ into the ER by the SERCA pump, which has two active Ca^2+^‐binding sites. *J*_out_ describes Ca^2+^ clearance from the cytoplasm through the plasma membrane via PMCA pumps that also have two binding sites.

In addition to these terms in the original Li & Rinzel model, we include the flux *J*_SOCE_, which describes the store‐operated Ca^2+^ entry pathway, filling the ER lumen with Ca^2+^ brought in at plasma membrane contacts. We do not resolve the detailed dynamics at the plasma membrane contacts but rather assume that SOCE entry operates in a rapid and regulated fashion to maintain a fixed concentration of free luminal Ca^2+^ at the contact sites (*c_cs_*). In this simplified model, we take the filling of the bulk ER lumen to proceed at a rate proportional to the difference between this fixed contact site concentration and the average luminal free Ca^2+^. The rate constant *k*_SOCE_ is extracted from our spatially resolved simulations, using the value at time *t* = 10 sec after refill is initiated. We note that this refill rate is already close to the long‐time plateau value (see Figure S11).

The rate of evolution for the free ER luminal Ca^2+^ is then found via the rapid‐binding assumption:

(13) $$\frac{du_{\mathrm{ER}}}{dt}=\frac{dc_{\mathrm{ER}}}{dt}\;/\left(1+\frac{K_{D}S}{{\left(u_{\mathrm{ER}}+K_{D}\right)}^{2}}\right)$$

### 6.7 Model Parameters

The half‐max binding concentrations for all regulatory sites on IP3R channels have been previously estimated, and we use the published values (see Table 2). The buffer binding strength *K_D_* and total capacity *S* has also been estimated from experimental data [15]. We fix the free Ca^2+^ level at contact sites based on an approximate measurement of ER free luminal Ca^2+^ in non‐firing cells (measured at 0.177 mm in WT iNeurons, Supplemental Figure S2E). The refill rate constant *k*_SOCE_ is determined from spatially resolved simulations, and the ER and cytoplasmic volumes *V*_ER_ *, V*_cyto_ are taken from our constructed WT network structures. Because we seek to isolate the effect of refill rate on Ca^2+^ oscillations, we use the same value of *V*_ER_ for both types of networks and alter only the refill rate constant. The remaining parameters: *v*_1_ *, v*_3_ *, v*_4_ *, k*_ER_ *, k*_PL_ *, a* are tuned to demonstrate the plausibility of refill‐rate‐dependent oscillations as shown in Figure 4.

**TABLE 2:** Model Parameters.

Symbol		Meaning	Value	Rationale
*d*_ip3_		IP3R IP3 site K*_d_* (no inhibition)	0.13 µm	[69]
*d*_2_		IP3R Ca^2+^ inhibition site K*_d_*	1.049 µm	[69]
*d*_3_		IP3R IP_3_ site K*_d_* (with inhibition)	943.4 nm	[69]
*d*_act_		IP3R Ca^2+^ activation site K*_d_*	82.34 nm	[69]
*k_D_*		Ca^2+^‐buffer protein dissociation constant	0.2 µm	[15]
*S*		Buffer protein site conc.	2.7 mm	[15]
*c*_cs_		Ca^2+^ at SOCE contact site	200 µm	current measurements
*V*_ER_		ER volume	212 µm^3^	model WT tubular network
*k*_SOCE_	(WT)	SOCE refill rate constant (WT)	6.5 µm^3^/s	spatial sims
*k*_SOCE_	(RTN3OE)	SOCE refill rate constant (RTN3)	4 µm^3^/s	spatial sims
[IP3]		IP3 concentration	0.4 µm	[50]
*v*_1_		Max IP_3_R flux rate	69 µm^3^/s	Tuned for amplitude
*v*_2_		Passive Ca^2+^ leak rate	0 µm^3^/s	Tuned
*v*_3_		SERCA uptake max rate	496 µm·µm^3^/s	Tuned
*v*_4_		Cytoplasmic clearance rate	368 µm·µm^3^/s	Tuned
*k*_ER_		SERCA half‐max Ca^2+^	0.117 µm	Tuned
*k*_PL_		Clearance half‐max Ca^2+^	0.106 µm	Tuned
*a*		IP3R Ca^2+^ inhibition rate const.	0.594 (µm · *s*)^−1^	Tuned

### 6.8 Implementation

The aspatial model described above was implemented in Matlab, with the variables *u*_ER_(*t*)*, c*_cyto_(*t*)*, h*(*t*) evolved forward in time using the built‐in ode45 function. Code for running this model is provided at https://github.com/lenafabr/aspatialCaERrefill.

## Author Contributions

E.A. conceived the study and designed and interpreted the experiments, V.D. designed, performed, analyzed, and interpreted the neuronal experiments and generated figures, P.P. analyzed the Ca^2+^ network data, designed and interpreted experiments, and provided advice on experimental protocols and on project development, E.K. and Y.Z. designed, conducted and interpreted the physical modelling, and wrote the modelling sections, T.K. performed the qPCR experiments, assisted with molecular biology and in cell experiments, provided advice on experimental protocols, and generated the graphical abstract, C.C. designed and performed calcium imaging experiments in COS7 and provided advice on project development, E.R.G. and R.P. designed and performed experiments in primary muscle cells, M.J.D. and J.F. designed and performed the field stimulation neurophysiological experiments, M.A. contributed to neuronal cell model generation, D.M. contributed to data generation and interpretation, J.C. contributed to development of concepts related to dementia‐associated FENIB neuroserpin and generation of material, V.D. and E.A. wrote the manuscript with critical help from D.M. and input from all the co‐authors.

## Conflicts of Interest

The authors declare no conflicts of interest.

## Supporting information




**Supporting File 1**: advs74905‐sup‐0001‐SuppMat.docx.

**Supporting File 2**: advs74905‐sup‐0002‐VideoS1.avi.

**Supporting File 3**: advs74905‐sup‐0003‐VideoS2.avi.

**Supporting File 4**: advs74905‐sup‐0004‐VideoS3.avi.

**Supporting File 5**: advs74905‐sup‐0005‐VideoS4.avi.

**Supporting File 6**: advs74905‐sup‐0006‐VideoS5.avi.

**Supporting File 7**: advs74905‐sup‐0007‐VideoS6.avi.

**Supporting File 8**: advs74905‐sup‐0008‐VideoS7.avi.

**Supporting File 9**: advs74905‐sup‐0009‐VideoS8.avi.

## Data Availability

The data that support the findings of this study are available from the corresponding author upon reasonable request.
