# Supplementary material for: Endoplasmic Reticulum Geometry Dictates Neuronal Bursting via Calcium Store Refill Rates and Exposes Selective Neuronal Vulnerability
Source: Adv Sci (Weinh). 2026 Mar 31;13(36):e21101. doi: 10.1002/advs.202521101 (PMC13317608; doi:10.1002/advs.202521101)
Supplement: Supplementary file 1 — Supporting File 1: advs74905‐sup‐0001‐SuppMat.docx. [file ADVS-13-e21101-s007.docx]

**Endoplasmic Reticulum Geometry Dictates Neuronal Bursting via Calcium Store Refill Rates**

Valentina Davi^1^, Pierre Parutto^1^, Yuyi Zhang^2^, Tasuku Konno^1^, Cecile Crapart^1^, Raquel Pereira^3,4^**,** John P Franklin^5,6^, Mosab Ali Awadelkareem^7^, Daniel Maddison^1^, Michael J Devine^5,6^, Edgar R. Gomes^3,4^, Joseph Chambers^3^, Elena Koslover^2^, Edward Avezov^1,*^

^1^UK Dementia Research Institute at University of Cambridge, Department of Clinical Neurosciences, Cambridge CB2 0AH, United Kingdom

^2^Department of Physics, University of California, San Diego, La Jolla, CA 92130

^3^GIMM - Gulbenkian Institute for Molecular Medicine, Avenida Prof. Egas Moniz, 1649-028 Lisboa, Portugal

^4^Faculdade de Medicina, Universidade de Lisboa, Av. Prof. Egas Moniz, 1649-028 Lisboa, Portugal

^4^Mitochondrial Neurobiology Laboratory, The Francis Crick Institute, London NW1 1AT, UK

^5^Department of Basic and Clinical Neuroscience, Institute of Psychiatry, Psychology and Neuroscience, King's College London, London SE5 8AF UK

^6^Department of Clinical and Movement Neurosciences, UCL Queen Square Institute of Neurology, University College London, London WC1N 3BG, UK

^5^Cambridge Institute for Medical Research (CIMR), Department of Medicine, University of Cambridge, The Keith Peters Building, Hills Road, Cambridge CB2 0XY, UK

^7^Nuffield Department of Clinical Neurosciences, University of Oxford, Oxford OX3 9DU

*Correspondence to [ea347@medschl.cam.ac.uk](mailto:ea347@medschl.cam.ac.uk)


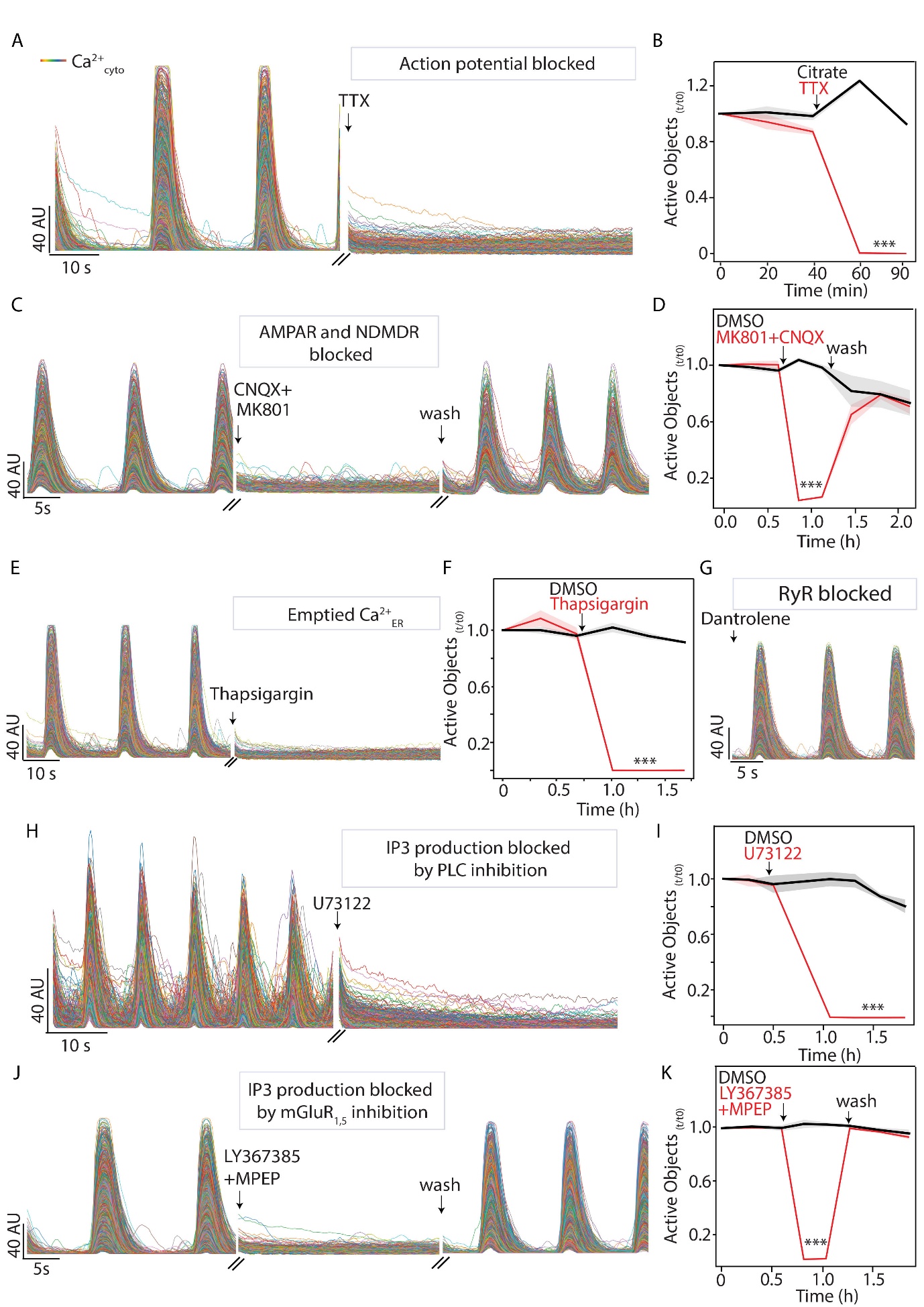


Figure S1: Characterisation of iNeurons’ Ca2+ bursts**A.** Single-cell cytosolic Ca^2+^ traces before and 1 min after TTX treatment (1 µM). **B.** Active cells (Active Objects) count over time in TTX and Citrate treated cultures. **C.** Single-cell cytosolic Ca^2+^ traces before, 1 min after CNQX and MK801 treatment (10 µM) and 5 min after washout. **D.** Quantification of (C) as in (B). **E.** Single-cell cytosolic Ca^2+^ traces treated with Thapsigargin (2 µM) as in (A). **F.** Quantification of (E) as in (B). **G.** Single-cell cytosolic Ca^2+^ traces 5 min after Dantrolene (10 µM). **H.** Single-cell cytosolic Ca^2+^ traces treated with U73122 (PLC inhibitor, 10 µM) as in (A). **I.** Quantification of (J) as in (B). **J.** Single-cell cytosolic Ca^2+^ traces treated with LY 367385 (100 µM) and MPEP (10 µM) as in (C). **K.** Quantification of (H) as in (B). In all the experiments in this figure, iNeurons were at Day 28 and cyto-Ca^2+^ was detected through Neuroburst. Red and black respectively represent treatment and treatment control. Statistical significance was determined by Student’s t test; *p<0.05, **p < 0.01, ***p<0.001, ****p<0.0001. Data are represented as mean (solid line) ± STD (shade) over n = 3 wells.


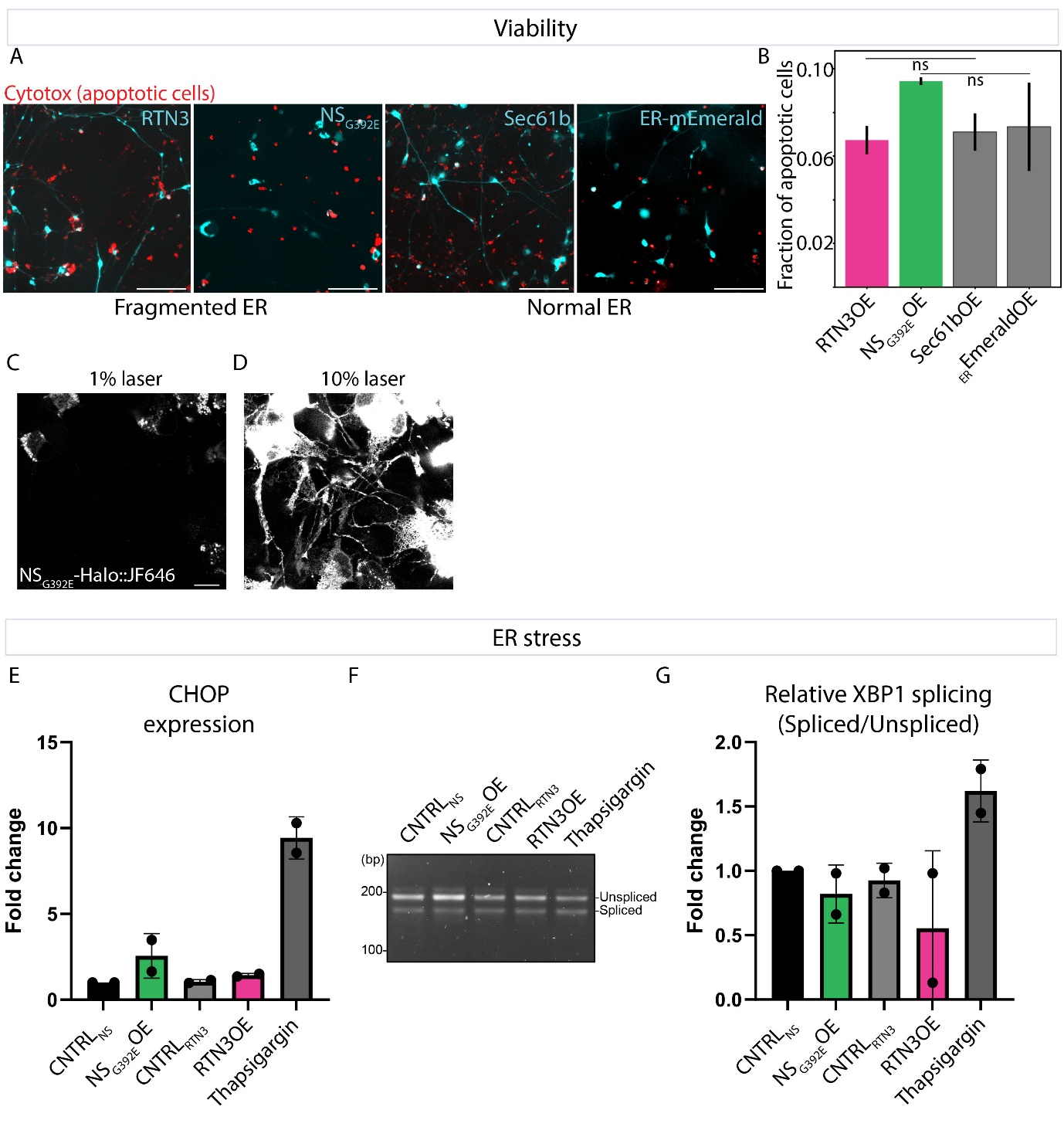


### Figure S2: Effect of ER fragmentation on iNeuron’s viability and ER stress

**A.** Micrographs of the apoptotic marker Incucyte^®^ Cytotox Red Dye (250 nM) in iNeurons after 7 days of exogenous expression of (from left to right) RTN3a-Halo, Halo-NS_G392E_, Halo-Sec61b (ER-membrane control) and mEmerald-KDEL (ER-lumen control). Halo was labelled with HaloTag Oregon Green Ligand. Scalebar: 100 μm. **B.** Fraction of cells with exogenous expressions as in (A) co-localising with the apoptotic marker (n = 3 wells, ^ns^p_RTN3_ = 0.65, ^ns^p_NSG392_ = 0.22). **C-D.** Micrograph of exogenously expressed Halo-NS_G392E_. Halo was stained with JF646 and the same field of view (FOV) was imaged with either 1% (**C**) or 10% (**D**) laser. Note the presence of Halo-NS_G392E_ in neurites. Scale bar: 10 μm**. E.** Real-time PCR analysis of UPR marker CHOP. Thapsigargin-treated iNeurons (0.5 μM, 5h) were used as a positive control of UPR upregulation by ER stress. Relative gene expression levels were calculated by ΔΔCt method using a house keeping gene (*GAPDH*) and sorted not-expressing cells (TMR negative) from the NS_G392_ infected sample as references. Two independent experiments were carried out. **F.** Representative agarose gel image of *XBP1* splicing assay. A partial *XBP1* sequence including splicing site was amplified from cDNA. **G.** Quantification of (**F**) analysing the ratio of spliced and unspliced product. The ratio (Spliced/Unspliced) was presented as a relative value to the sorted not-expressing population (TMR negative) from the NS_G392_ infected sample.

###
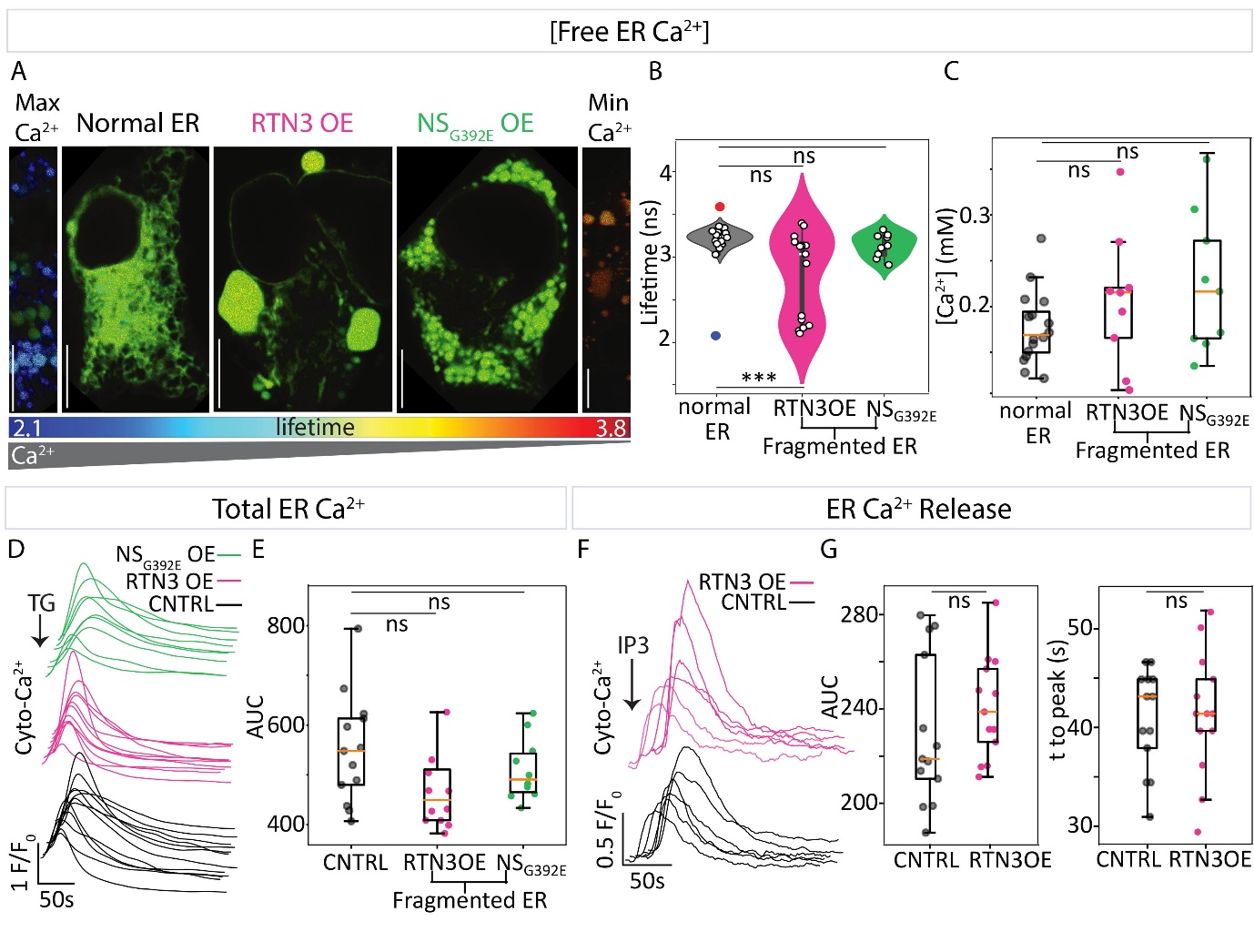


### Figure S3: Fragmented ER maintains its ability to store and release Ca^2+^

**A-C.** Measurements of free ER Ca^2+^ concentration. **A.** Fluorescence lifetime imaging microscopy (FLIM) images of ER-targeted D4ER-Tq Ca^2+^ FRET probe in iNeurons. From left to right: maximal ER Ca^2+^ obtained by ionomycin treatment (10 μM), WT, RTN3aOE, NS_G392E_OE, minimal ER Ca^2+^ obtained by Thapsigargin treatment (3 μM). Scalebars: 5 µm. **B.** FLIM average lifetimes detected as in (A) of WT (n = 16 cells), RTN3aOE (n = 15 cells, ^ns^p_top_ = 0.28, ***p_bottom_ = 1.33 x 10^-16^) and NS_G392E_OE (n = 9 cells, ^ns^p = 0.06) iNeurons. Each dot represents the lifetime from a single cell. Lifetimes in maximal and minimal ER Ca^2+^ conditions are indicated as red and blue dots respectively. **C.** Luminal free [Ca^2+^]_ER_ calculated from values in (D) of WT (n = 16 cells), RTN3aOE (n = 9 cells, ^ns^p = 0.20) and NS_G392E_OE (n = 9 cells, ^ns^p = 0.053) iNeurons. Each dot represents the [Ca^2+^]_ER_ from a single cell. The low-lifetime population of RTN3OE iNeurons was excluded as an artifact was suspected in this subpopulation of cells presenting extra-large vesicles. **D-E.** Measurement of Total ER Ca^2+^ load. **D.** Single-cell cytoplasmic Ca^2+^ traces detected by Cal-520 after ER Ca^2+^ release elicited by 3 μM Thapsigargin in WT (black), RTN3 OE (pink) and NS_G392E_OE (green) iNeurons. **E**. Integrated fluorescence intensity (Area Under the Curve – AUC) of traces in (D) of WT (n = 13 cells), RTN3 OE (n = 12 cells, ^ns^p = 0.22) and NS_G392E_OE (n = 10 cells, ^ns^p = 0.31) iNeurons. Each dot represents the AUC from a single cell. **F.** Single-cell cytoplasmic Ca^2+^ traces detected by GCaMP8 after ER release through light-induced IP3 uncaging in whole field of view containing WT (black) and RTN3 OE (magenta) iNeurons. **G.** Integrated fluorescence intensity (AUC, left, ^ns^p=0.35) and time to peak (right, ^ns^p=0.82) of WT (n = 13 cells) and RTN3 OE (n = 13 cells) iNeurons. Each dot represents AUC and time-to-peak from a single cell. Statistical significance was determined Student’s t test.

###
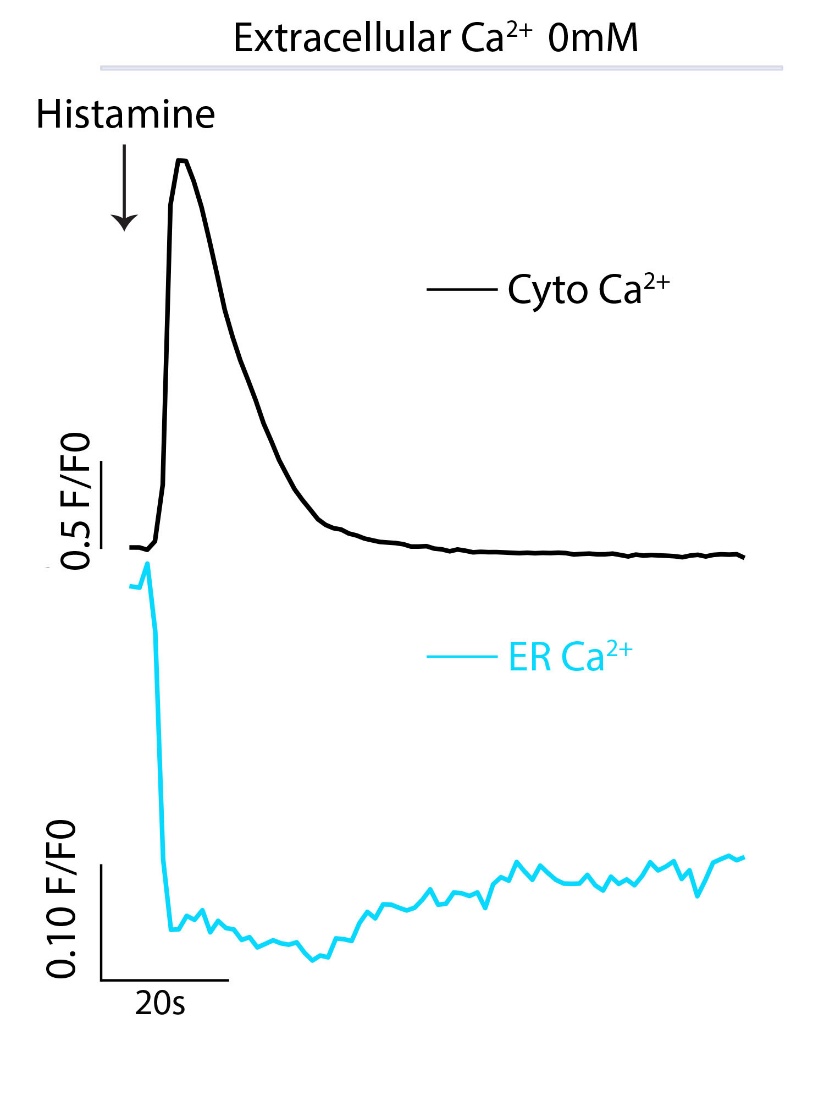


### Figure S4: ER does not re-uptake cytosolic Ca^2+^

Single-cell cytosolic Ca^2+^ (black) and ER Ca^2+^ (cyan) traces simultaneously detected in COS7 cells through GCaMP8 and R-CEPIA_ER_ respectively. Arrow represents time of treatment with Histamine (100 µM). EGTA (3 mM) was added to the media 5 mins before the experiment.

###
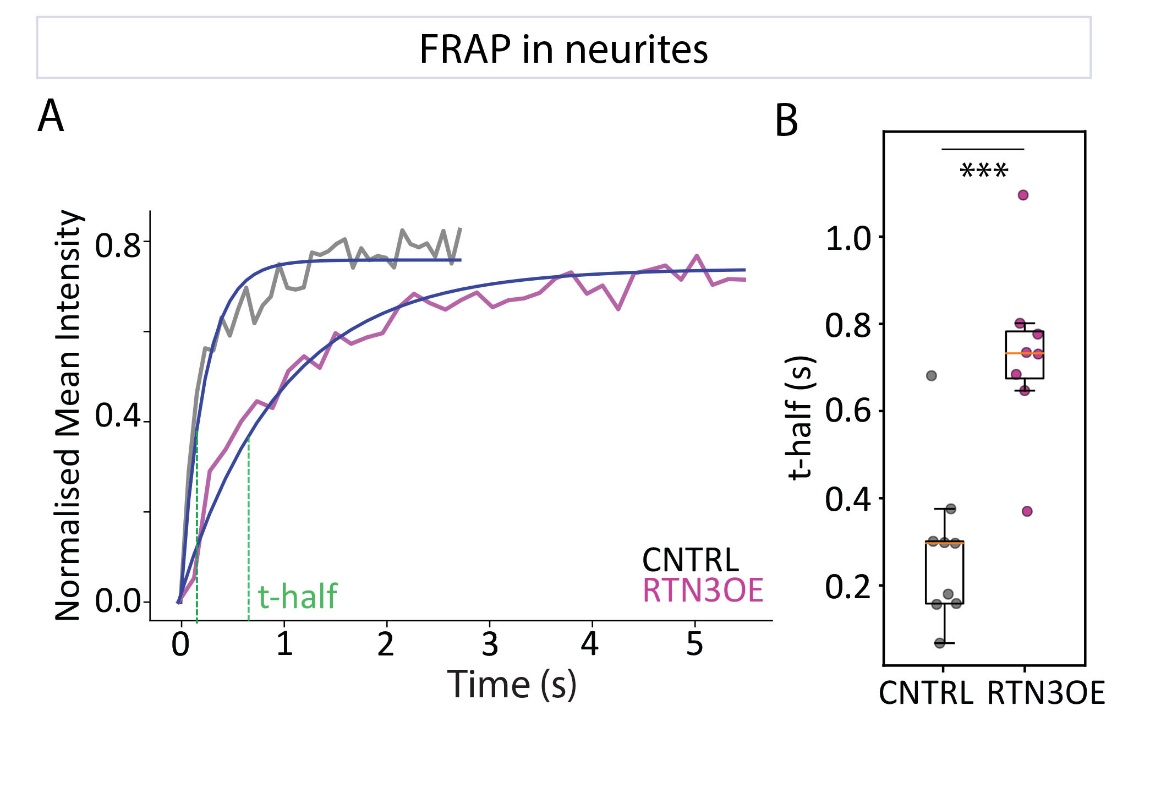


### Figure S5: ER fragmentation affects luminal connectivity in neurites

**A.** ER luminal protein mEmerald-KDEL intensity traces after photobleaching (grey: control (not infected), magenta: RTN3aOE). Blue curve: exponential fit, green dashed line: half recovery time (t-half). **B.** Half recovery time (t-half) values from exponential fits as in (A) from WT (n = 9 cells) and RTN3 OE (n = 8 cells, ***p = 1.9 x 10^-4^). Each dot represents the t-half from a single cell. Statistical significance was determined Student’s t test.

###
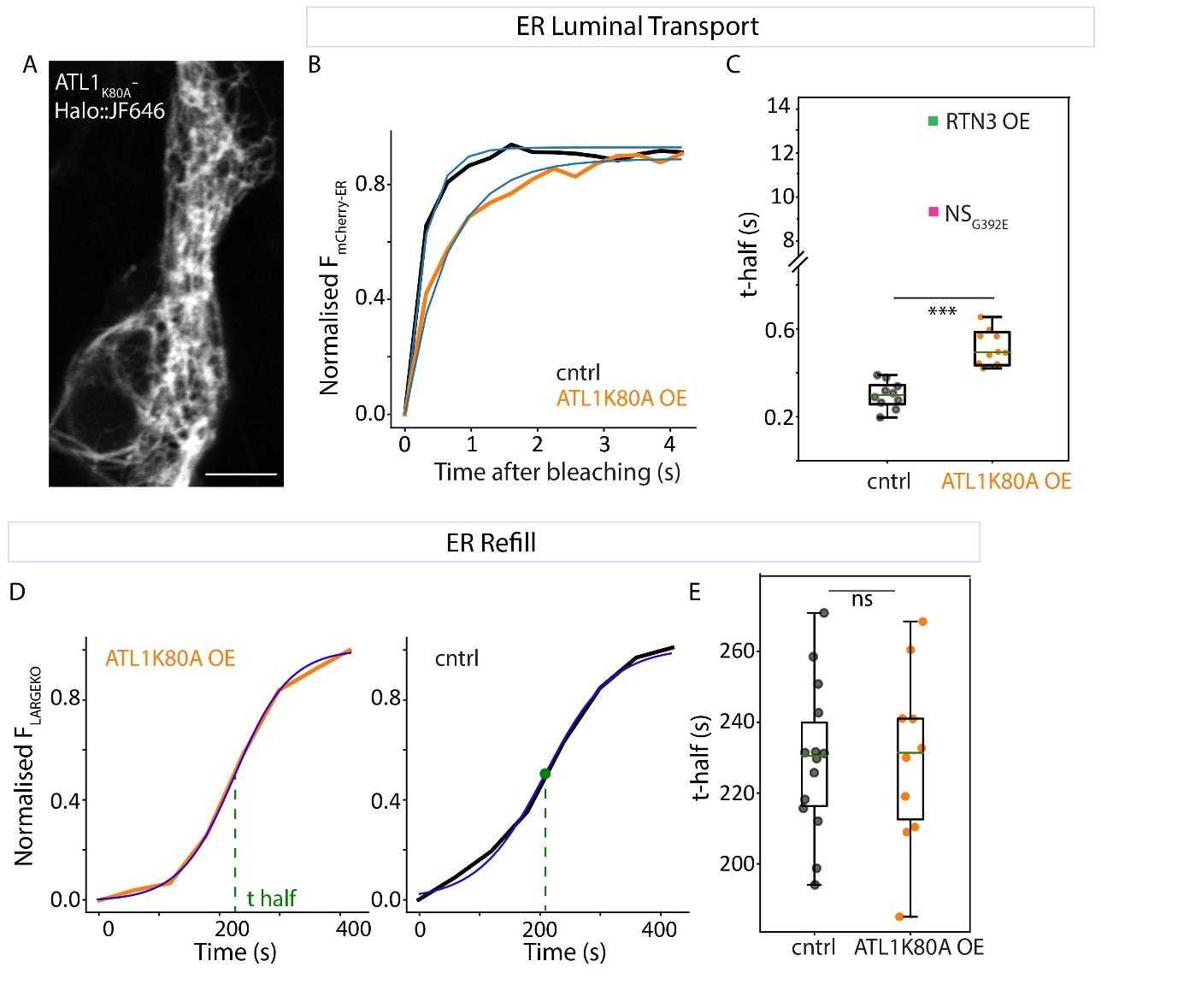


### Figure S6: ATL1 K80A modestly slows ER luminal transport without affecting refill

**A.** Micrograph of the ER in iNeurons with exogenously expressed ATL1 K80A-Halo labelled with HaloTagJF646. Scale bar: 5 µm. **B.** mCherry-KDEL intensity traces after Fluorescence Recovery After Photobleaching (FRAP) performed in iNeurons (black: control (not infected), orange: ATL1 K80A OE). Blue curve: exponential fit. **C.** Half recovery time (t-half) values from the exponential fit as in (B) from WT (n = 10 cells) and ATL1 K80A OE (n = 10 cells, ***p = 1.60 x 10^-6^). Each dot represents the t-half from a single cell. Squares represent the median t-half recorded from RTN3aOE (pink) and NS_G392E_ (green). **D.** Single-cell ER Ca^2+^ traces of control (not infected, black) and ATL1 K80A OE (orange) detected with the ER Ca^2+^ sensor LAR-GECO after washout of BTP2 (ORAI1 blocker, 10 µM, 20 min incubation). Blue curve: sigmoid fit, green dashed line: time to half-recovery (t-half). **E.** Half recovery time (t-half) values obtained from sigmoid fits as in (D) of control (not infected, n = 14 cells) and ATL1 K80A OE (n = 10 cells, p = 0.97). Each dot represents the t-half from a single cell. Statistical significance was determined Student’s t test.

###
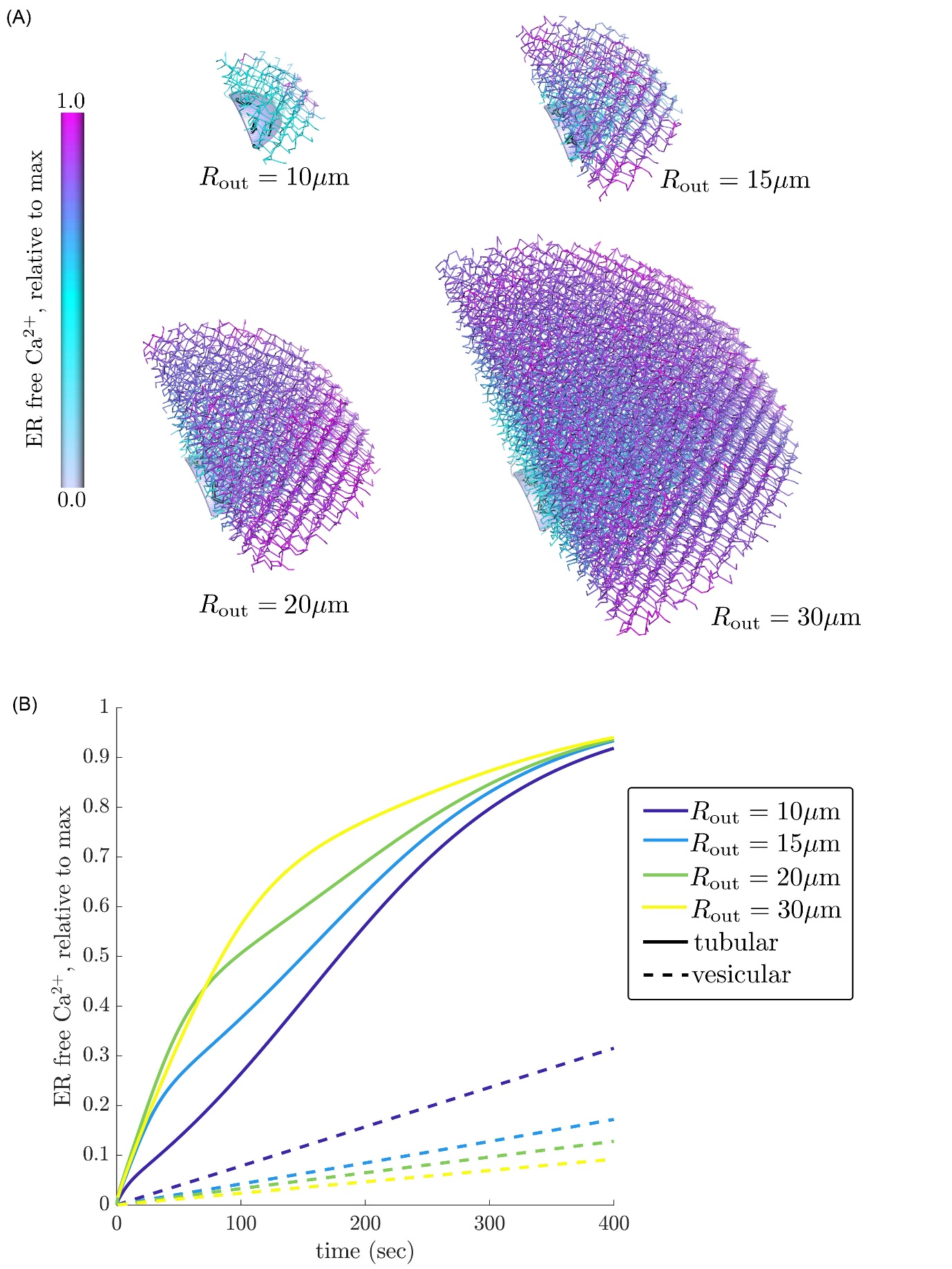


### Figure S7: Refilling ER networks of increasing spatial size.

**A.** Networks are constructed in a domain with outer radius R_out_ = 10-30um, while the inner domain radius, perinuclear ER volume, and surface density of PM contact sites remain constant. For computational efficiency, only one octant sector for the spherically symmetric system is modelled. Sample snapshots for each tubular network structure are shown at 2min after refill begins, with colour corresponding to free Ca^2+^.  **B.** The average cumulative free Ca^2+^ in the network is plotted over time for tubular networks (solid lines) and vesiculated structures with bubble radius R=0.6µm (dashed lines). Larger tubular networks have a greater fraction of their volume in the outer periphery and are initially faster to fill. However, after the initial transient, the steady-state period during which the perinuclear region is filling exhibits a lower refill slope for large tubular networks. Vesicular networks are limited by filling vesicles in the peripheral zone and thus exhibit slower overall refill as the total network size increases. The distinction in refill rate between tubular and vesicular networks becomes more extreme for larger cell sizes.


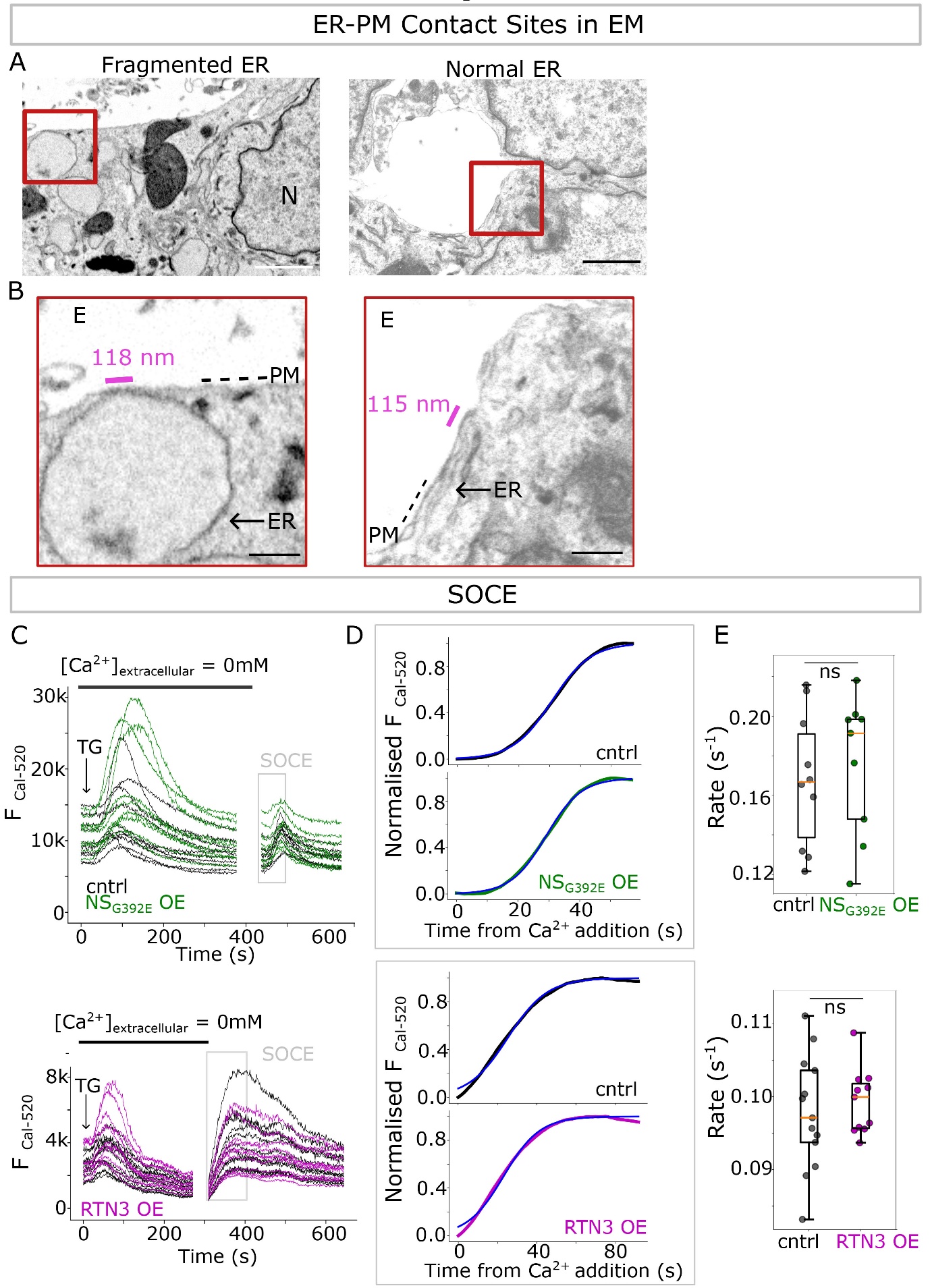


### Figure S8: ER fragmentation does not alter ER-PM contact sites width and SOCE

**A.** Scanning electron micrograph of iNeurons with deformed ER via exogenous expression of NS_G392E_ (left) and normal ER (right). N = nucleus. Scale bars: 1 µm. **B.** Blow-up of red squares in (A) displaying ER-PM contacts (marked in pink). E = extracellular space. Scale bars: 250 nm. **C-E.** Evaluation of Store Operated Calcium Entry (SOCE). **C.** Single-cell cytosolic Ca^2+^ traces detected through Cal-520 in iNeurons exogenously expressing Halo-NS_G392E_ (green), RTN3 (magenta) or control iNeurons (not transduced, black) from same FOV. In absence of extracellular Ca^2+^ (achieved by 5 minutes pre-incubation with 3mM EGTA), Thapsigargin (TG) is added to block SERCA and thus empty the ER (first spike), then extracellular Ca^2+^ is re-introduced leading to SOCE (second spike, grey square). **D.** Blow-out of one representative normalised and smoothed cytosolic Ca^2+^ trace from (C) during SOCE (grey square in (C)). See Methods for smoothing and normalisation. Blue curve: sigmoid fit. **E.** Logistic growth rate values obtained from sigmoid fits as in (D) of not expressing control from FOV of NS_G392E_ experiment (not infected, n = 10 cells), Halo-NS_G392E_OE (n = 9 cells, p = 0.08), not expressing control from FOV of RTN3 experiment (n = 13 cells), and RTN3a OE (n = 11, p = 0.57). Each dot represents the t-half from a single cell. Statistical significance was determined Student’s t test.

###
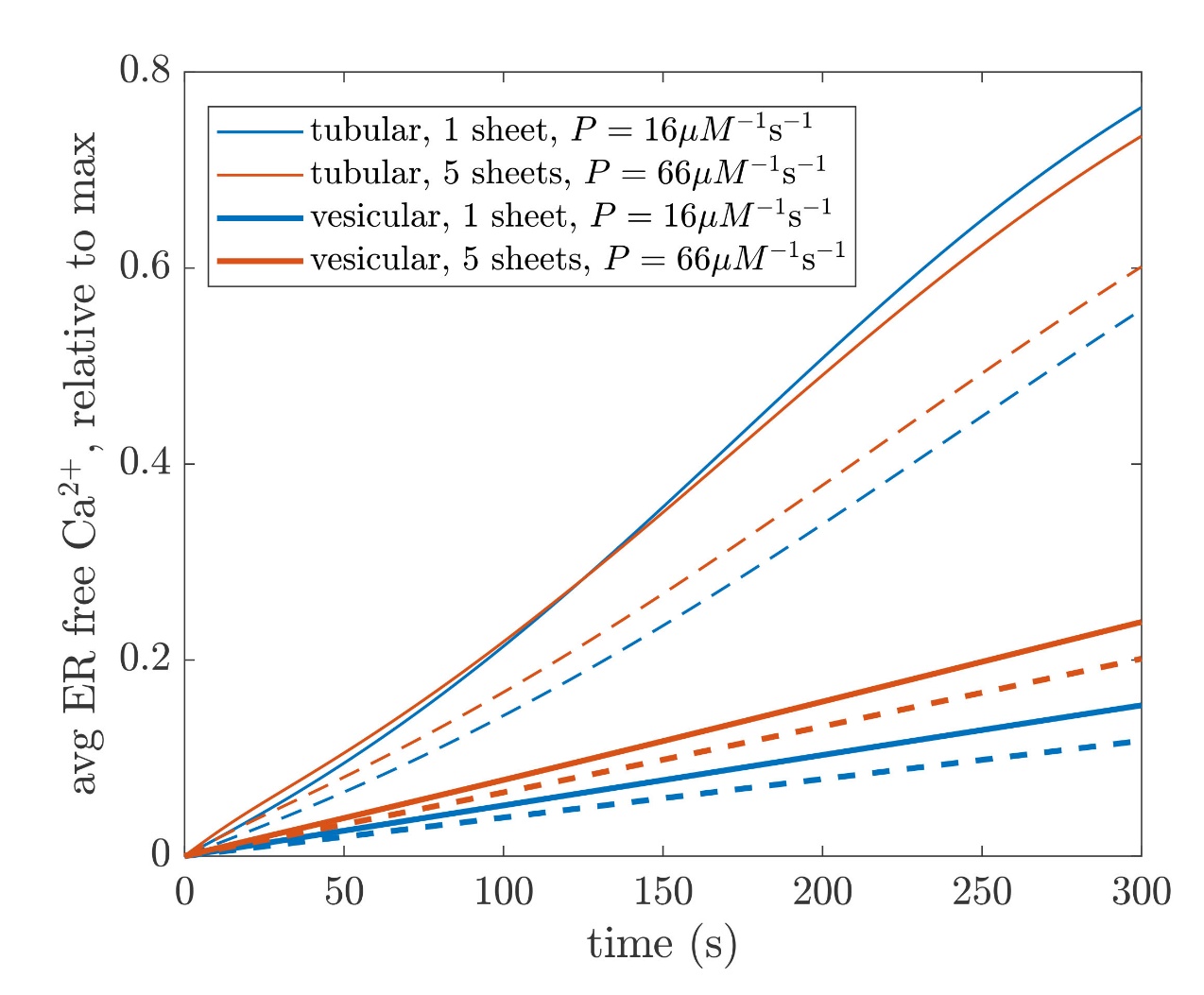


### Figure S9: ER refill dynamics for models with different rates of local Ca^2+^ entry through SOCE

The SOCE rate constant P is adjusted to give a refill half-time of 200sec for the tubular network. Two different perinuclear volumes are considered, with 1 sheet (blue) or 5 sheets (red) wrapped around the nucleus. Higher perinuclear volumes require more rapid SOCE to give the same refill half-time. The value P=16µM^-1^s^-1^ corresponds to a lower bound. For each case, the SOCE rate constant P is reduced by 30% (dashed curves), demonstrating the moderate decrease in overall refill rates. Thick lines show refill for a vesiculated network, with the same values of P and perinuclear volumes. Note that the change in network structure has a much greater effect on refill rates than the decrease in P.

###
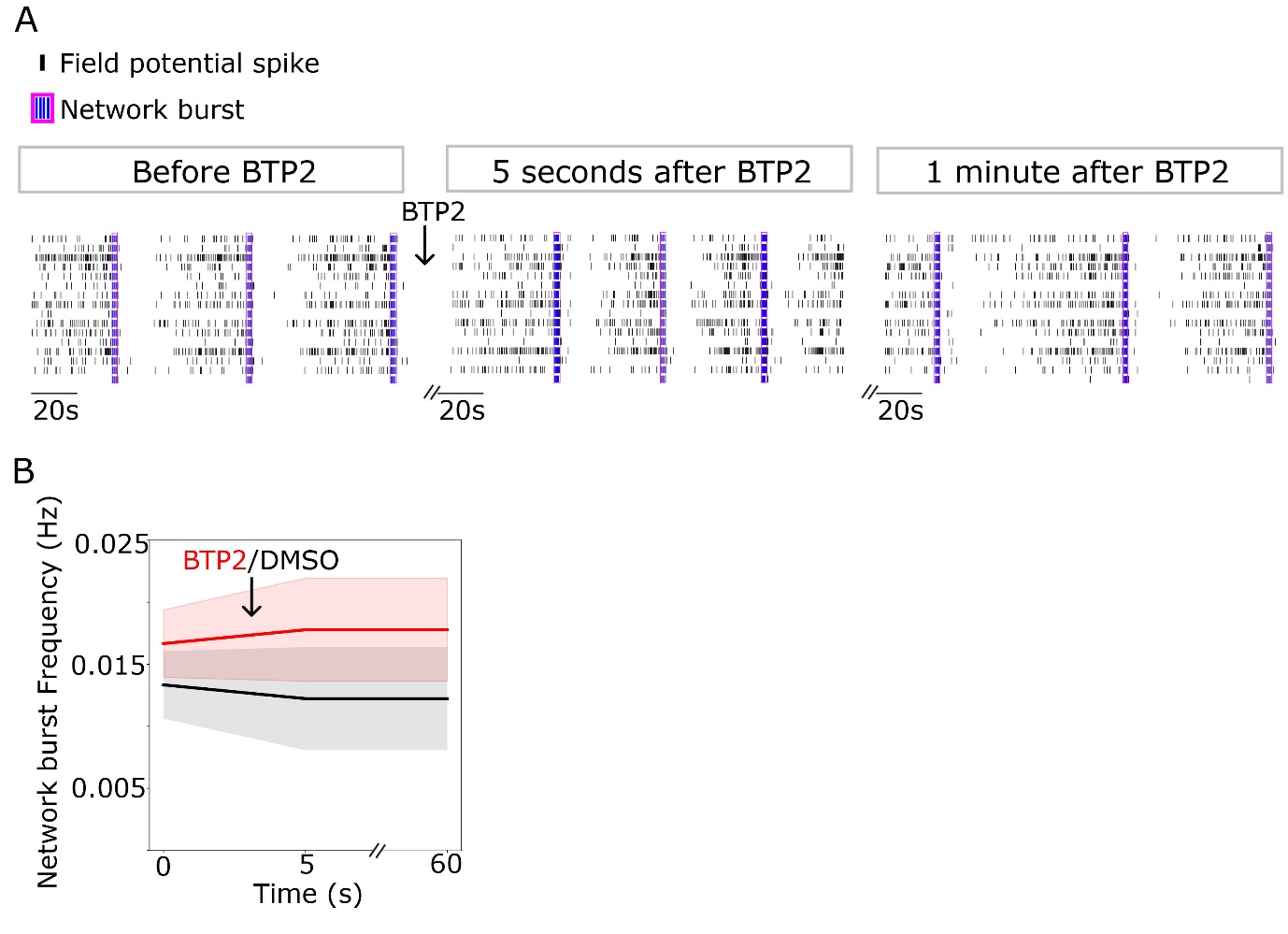


### Figure S10: Blocking ORAI1 via BTP2 does not affect field potentials

**A.** Temporal raster plot of detected spikes from multiple electrodes of a Multi-Electrodes Array (MEA) over time in Day 28 iNeurons, before and after treatment with BTP2 (ORAI1 blocker, 10 µM). **B.** Network burst frequency over time from MEA recordings as in (A) (n = 3 wells, p = 0.25).

###
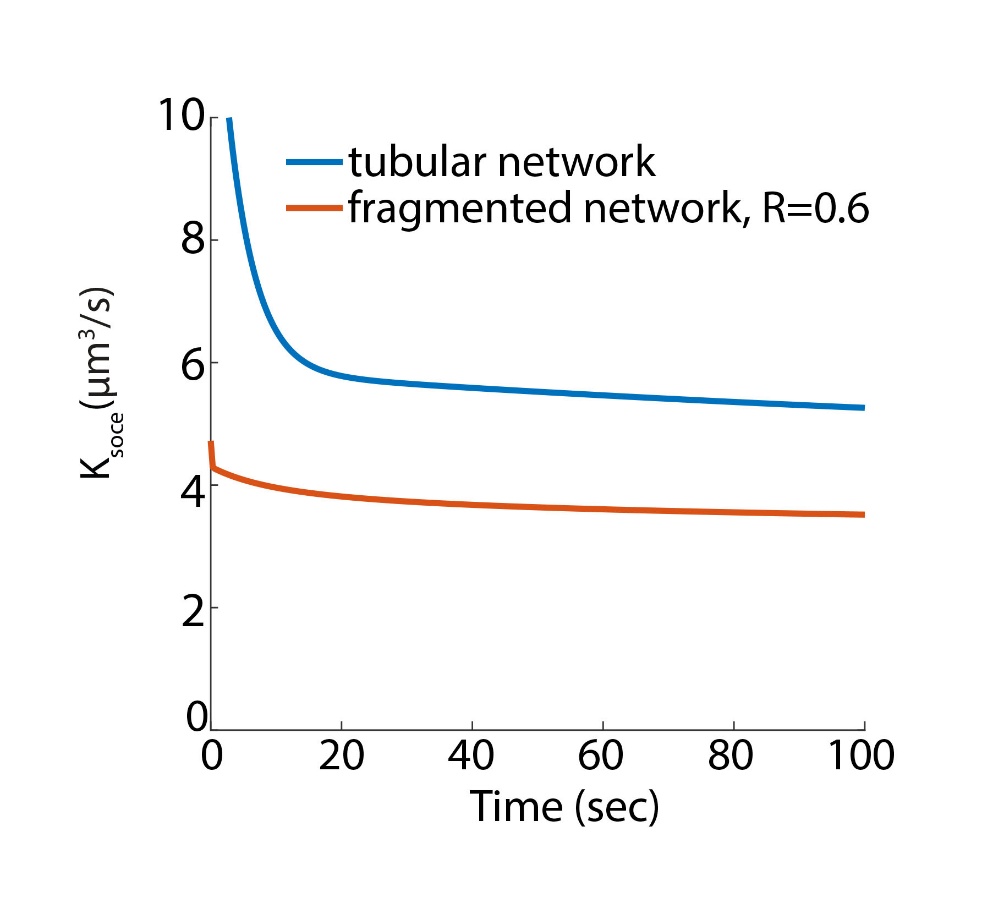


### Figure S11: Modelled refill rates

Effective refill rate k_SOCE over time for a tubular network (WT) and a network of spheres with radius 0.6 µm (RTN3OE), extracted from 3D spatial simulations. Both networks have 40 peripheral contacts with the extracellular environment. The rate is initially high during peripheral ER filling and gradually plateaus as the peripheral network saturates and the reservoir continues to fill. We use the value of kSOCE at 10 seconds—6.5 µm³/s for WT and 4.0 µm³/s for RTN3OE—as input parameters for the aspatial model to reflect the quasi-steady-state refill rate.

###
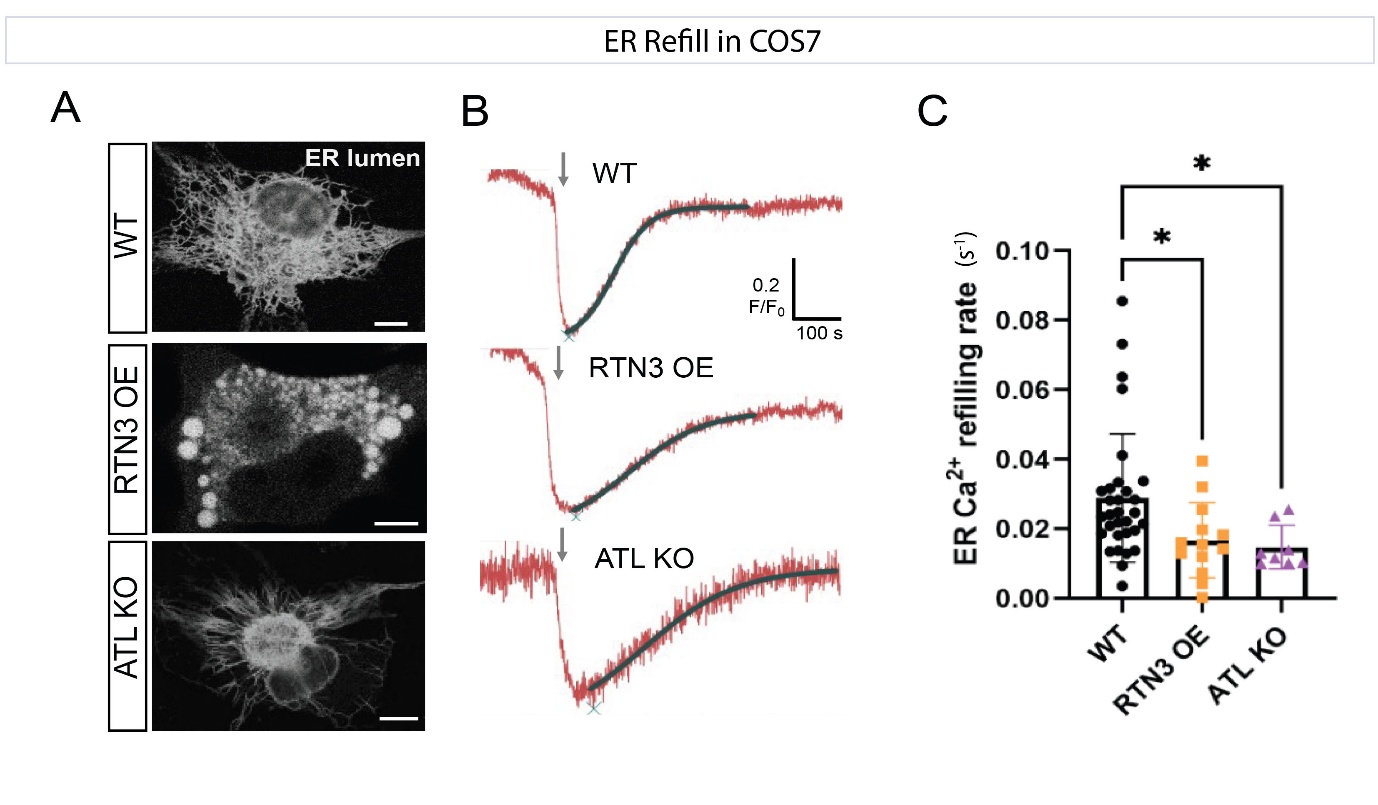


### Figure S12: ER Ca^2+^ refill is slower in ATL2/3 KO ER networks in COS7 cells.

**A.** Micrographs of the ER lumen (visualised through R-CEPIA) in COS-7 cells WT (top), RTN3 OE (middle) and ATL2/3 KO (bottom). Scale bars: 10 µm. **B.** Normalized intensity profiles of an ER Ca^2+^ probe’s signal (ER-LAR-GECO1) over time in live COS-7 cells WT (top), RTN3 OE (middle) and ATL2/3 KO (bottom). Histamine (100 µM) addition is indicated by the arrow. Cytosolic Ca^2+^ was buffered with Bapta-AM (100 µM) to promote spatial globalisation of Ca^2+^ release. **C.** Quantification of ER Ca^2+^ refilling rates, obtained from fitting the recovery portion of the intensity profiles (sigmoid fits indicated by dark green lines in (B)). Each dot represents the refill rate for a single cell (WT: n= 31, RTN3 OE: n=13, ATL2/3 KO: n=8 cells. *p < 0.05, KW test).

###
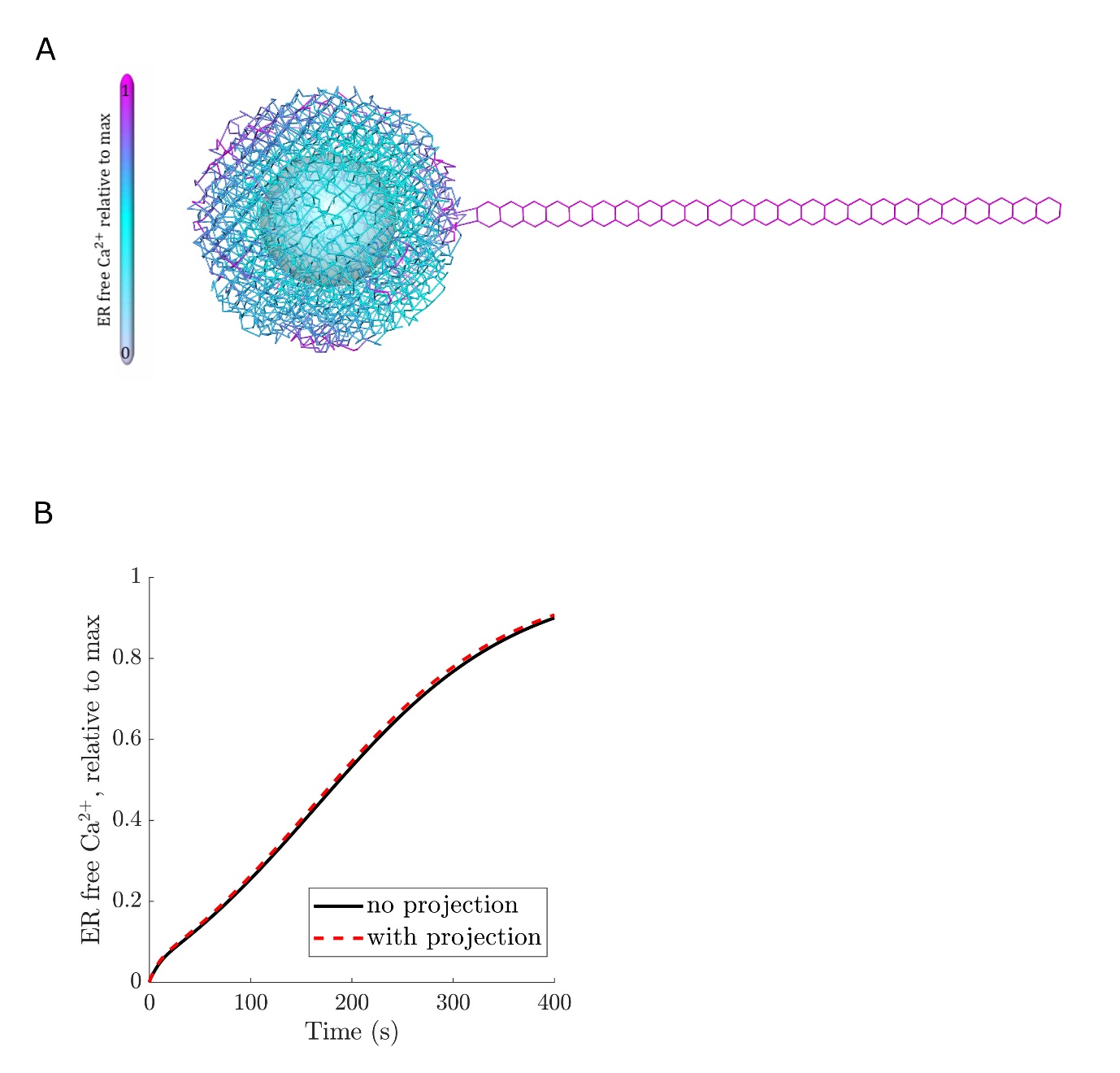


### Figure S13: Refill of simulated ER networks is not sensitive to the presence of long projections.

**A.** A projection of 43 µm length is appended to the tubular ER network in Fig. 3G. The ER within the projection is treated as parallel tubules with cross-links every 2 µm. A fraction of nodes in the projection is selected as PM contact sites, yielding a PM contact surface density identical to that in the soma. Simulation snapshot of local free Ca^2+^ concentration is shown at time 2 min after refill begins. Note that the projection is filled much more rapidly than the bulk ER network in the soma. **B.** Cumulative free Ca^2+^ averaged over full ER structure is plotted versus time, showing little difference with (red) and without (black) the projection.

Video 1. iNeurons’ spontaneous synchronous cytosolic Ca^2+^ bursts, related to Figure 1C. Time-lapse imaging of cytosolic Ca^2+^ (detected through Neuroburst) in iNeurons after 28 days of differentiation.

Video 2. iNeurons’ cytosolic Ca^2+^ bursts depend on ER Ca^2+^, related to Figure 1D. Time-lapse imaging of cytosolic Ca^2+^ in iNeurons as in Video1, before (left) and after CPA treatment (25 µM, middle), and after washout (right). During CPA treatment - causing ER Ca^2+^ depletion - the synchronous cytosolic bursts stop.

Video 3. ER fragmented through RTN3aOE can’t support the iNeurons’ cytosolic Ca^2+^ bursts, related to Figure 2E-G. Time-lapse imaging of cytosolic Ca^2+^ of iNeurons as in Video1 (middle), with sparse exogenous expression of RTN3a-Halo (labelled with Halo-ligand JFX650, magenta, left). iNeurons expressing RTN3-Halo don’t fire.

Video 4. ER fragmented through NS_G392E_OE can’t support the iNeurons’ cytosolic Ca^2+^ bursts, related to Figure 2E-G. Time-lapse imaging of cytosolic Ca^2+^ of iNeurons as in Video1 (middle), with sparse exogenous expression of Halo-NS_G392E_ (labelled with Halo-ligand JFX650, magenta, left). iNeurons expressing Halo-NS_G392E_ don’t fire.

Video 5. Loss of bursts synchrony after ER fragmentation, related to Figure 2H-J. Time-lapse imaging of cytosolic Ca^2+^ of iNeurons as in Video1, before (left) and after (middle) abundant exogenous expression of RTN3a-Halo (labelled with Halo-ligand JF646, magenta, right). After expressing RTN3a-Halo, the iNeurons’ network loses synchrony.

Video 6. ER fragmentation affects ER Ca^2+^ refill, related to Figure 3D-F. Time-lapse imaging of ER Ca^2+^ in iNeurons detected through GCaMP6ER-150 (left) after emptying of ER Ca^2+^ through BTP2 (10 µM) and washing it out. The iNeuron expressing RTN3a-Halo (labelled with Halo-ligand JF646, right) recover its ER Ca^2+^ slower than the one not expressing it. Timestamp is in format mins : secs. Scalebar: 20 µm.

Video 7. iNeurons’ cytosolic Ca^2+^ bursts depend on ER refill through SOCE, related to Figure 4A. Time-lapse imaging of cytosolic Ca^2+^ in iNeurons as in Video1, before and after BTP2 treatment (10 µM). After adding BTP2 – which inhibits SOCE - the synchronous cytosolic bursts gradually decrease in amplitude until completely stopping.

Video 8. Cytosolic calcium dynamics in primary skeletal muscle cells during electrical field stimulation (10V, 1Hz), related to Figure 5. Timelapse movie showing primary skeletal muscle cells, differentiated for 7 days, and expressing the GCaMP calcium reporter. Arrow points to a cell that was overexpressing Rtn3. Scale bar 10 μm.
